# Signatures of geography, climate and foliage on given names of baby girls

**DOI:** 10.1017/ehs.2022.53

**Published:** 2022-11-25

**Authors:** Raymond B. Huey, Donald B. Miles

**Affiliations:** 1 Department of Biology, University of Washington, Seattle, WA, USA; 2 Department of Biological Sciences, Ohio University, Athens, Ohio, USA

**Keywords:** Given names, onomastics, seasonality, climate influence on culture

## Abstract

Parents often weigh social, familial and cultural considerations when choosing their baby's name, but the name they choose could potentially be influenced by their physical or biotic environments. Here we examine whether the popularity of month and season names of girls covary geographically with environmental variables. In the continental USA, April, May and June (Autumn, Summer) are the most common month (season) names: April predominates in southern states (early springs), whereas June predominates in northern states (later springs). Whether April's popularity has increased with recent climate warming is ambiguous. Autumn is most popular in northern states, where autumn foliage is notably colourful, and in eastern states having high coverage of deciduous foliage. On a continental scale, Autumn was most popular in English-speaking countries with intense colouration of autumn foliage. These analyses are descriptive but indicate that climate and vegetation sometimes influence parental choice of their baby's name.

## Graphical abstract

**Figure:**
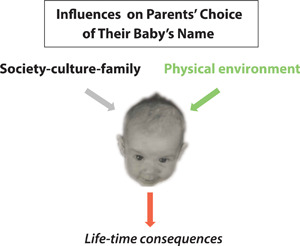

**Social media summary:** Geographic patterns of popularity of month and of season names suggest that physical and biotic environments can influence choice of baby name.

## Introduction

Babies begin life with a given name chosen by their parents. That choice can be influenced by the parents’ own social experiences, family relationships and cultural sensitivity (Schlesinger, 1941; Lieberson & Bell, 1992; Fryer & Levitt, 2004; Twenge et al., 2016) or even by a name's sound symbolism (Berger et al., 2012; Pitcher et al., 2013; Suire et al., 2019). The chosen name – whatever prompted its choice – can have life-long effects on the offspring's self-identity (Allport, 1937) and achievements (Moss-Racusin et al., 2012; Goldstein & Stecklov, 2016), as well as on its exposure to stereotyping and prejudice (Bertrand & Mullainathan, 2004; Moss-Racusin et al., 2012; Okonofua & Eberhardt, 2015).

Some names are perennially and broadly popular (Newberry & Plotkin, 2022), but others show striking boom and bust patterns of popularity (Lieberson & Bell, 1992; Berger & Le Mens, 2009; Liu et al., 2022; Newberry & Plotkin, 2022) (Figure S1). Still other names vary geographically in popularity, suggesting that regional cultural patterns can influence name choice (Varnum & Kitayama, 2011; Bentley & Ormerod, 2012; Barucca et al., 2015; Pomorski et al., 2016; Liu et al., 2022).

**Figure 1.**
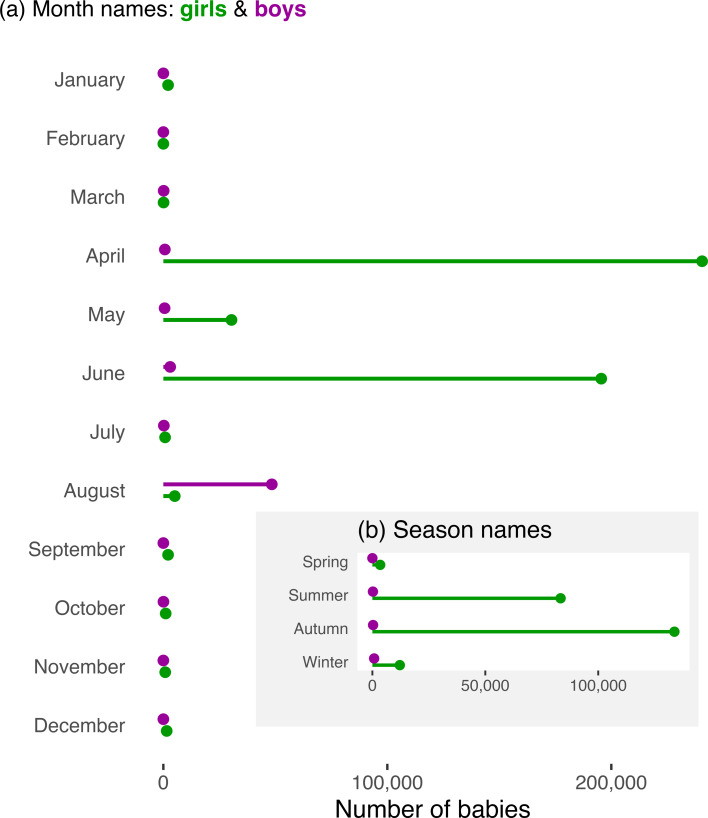
Numbers of babies with month or season names in the USA (1910–2021). (a) Total numbers of girls and boys with each month name. April, May and June are most common for girls, whereas August is the only common name for boys. (b) Season names are much more common for girls than boys, and Autumn and Summer are the most common season names for girls.

Climate is a composite environmental factor that has diverse effects on human systems and culture (van de Vliert, 2013; Carleton & Hsiang, 2016) and might influence name choice, either directly or via its influence on the local biota and its phenology. This climatic influence has, however, rarely been examined (Berger et al., 2012; He et al., 2021). Here we evaluate whether climatic and biotic environments influence name choice on geographic scales. We focus on calendar names (of months and seasons), which link directly to environmental seasonality. April, May and June are by far the most common month names for baby girls (Figure 1a). These months are associated with spring-like weather and vegetive renewal in the Northern Hemisphere and symbolise new life, youth and bounty, as in traditional English novels and poems (Supplementary Materials 1). If popularity of these names is linked to climate, then the relative frequency of each month name should shift geographically in concert with the timing of spring weather. By extension, the relative frequency of season names (e.g. Autumn) should shift geographically with seasonality of climate and especially with the intensity of colouration of deciduous foliage in autumn.

**Figure 2.**
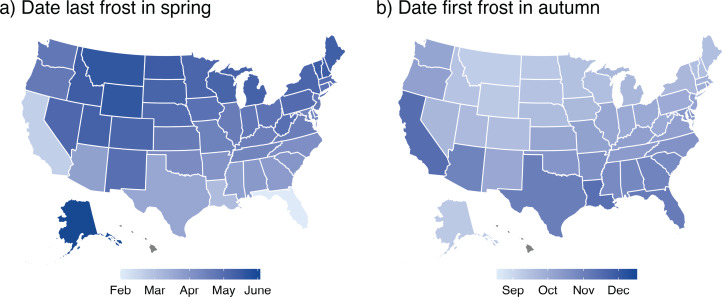
Onset of spring and autumn in USA. (a) Onset of spring is indexed by the median date of the last frost (by state) in spring by state. (b) Onset of autumn is indexed by the median date of the first frost in autumn. [Note: populated regions of Hawaii do not experience frosts.]

We focused on the relative frequencies of month and of season names (by state) of baby girls in the USA, because boys are rarely given calendar names (Figure 1). We proposed two hypotheses before inspecting or compiling data. First, because spring-like weather arrives earlier in southern than in northern states (Figure 2a), April should be relatively more common in the south, whereas May or June should be most common in the north. Second, because recent climate warming has advanced the onset of spring and associated phenology (Körner & Basler, 2010), April should be increasing in frequency (relative to May and June). Despite making these *a priori* predictions, we were initially concerned that any environmental effects on names might be swamped by cultural fads in name popularity (Lieberson & Bell, 1992; Berger & Le Mens, 2009; Newberry & Plotkin, 2022) (Figures S1 & S2) and drift (Hahn & Bentley, 2003), as well as by population shifts and immigration (Rogerson & Kim, 2005). Nevertheless, we proceeded to obtain and analyse name data.

Soon after we began analysing month names, we realised that we could also evaluate whether season names (e.g. Spring) showed geographic trends. However, when we found that Autumn (not Spring) was the most common season name (Figure 1b), but still before quantifying potential geographic patterns, we predicted that Autumn would be relatively frequent in high-latitude states, where autumn arrives relatively early (Figure 2b) and where deciduous foliage is most intensely coloured in autumn (Liu et al., 2017).

As with all large-scale analyses of baby names (Bentley & Ormerod, 2012; Barucca et al., 2015; Liu et al., 2022; Newberry & Plotkin, 2022), our analyses are descriptive. However, most hypotheses we evaluate here were derived *a priori* and were based on environmental factors that vary geographically and that potentially influence name choice. Our resulting investigations fit within a general theoretical framework emphasising the sensitivity of human culture to the environment (van de Vliert, 2009; Carleton & Hsiang, 2016).

## Materials and methods

### Sources of month and season names

We accessed baby name data (*N* = 351.8M, 1910–2021) for the USA from the Social Security Administration (Supplementary Materials 2). ‘National files’ (1910–2021) report year of birth, assigned sex, given name and number of occurrences of each name. ‘State-specific’ files also indicate state of birth (1910–2021). To maintain privacy the Social Security Administration excludes names that occurred fewer than five times in a file. Data sources for name frequency in some other English-speaking countries, as well as sources of environmental and related data (e.g. coordinates of population centres of states, mean altitude, coverage of deciduous trees and shrubs), are available (Supplementary Materials 3 and 4).

### Spatial analyses

**Figure 3.**
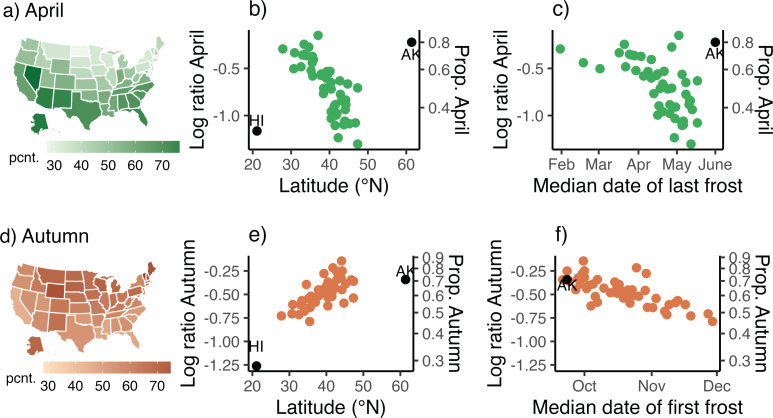
Geographic and climatic trends of relative frequency of April and of Autumn names of girls (1910–2021). (a) Choropleth map of relative percentage of all girls with ‘spring-month’ names that are named April. (b) Log ratio of April (equivalent proportion is on right *y*-axis) is negatively correlated (see text) with population-centred latitude of each CONUS state (i.e. states in continental USA). Points for Alaska (AK) and Hawaii (HI) are highlighted. (c) Log ratio of April is inversely correlated with median date of last frost in spring. [Note: Hawaii does not experience frosts and is absent from panels (c) and (f).] (d) Map of relative proportion of girls with season names that are named Autumn. (e) Log ratio of Autumn is positively correlated with latitude and (f) inversely correlated with the date of the first frost in autumn.

**Figure 4.**
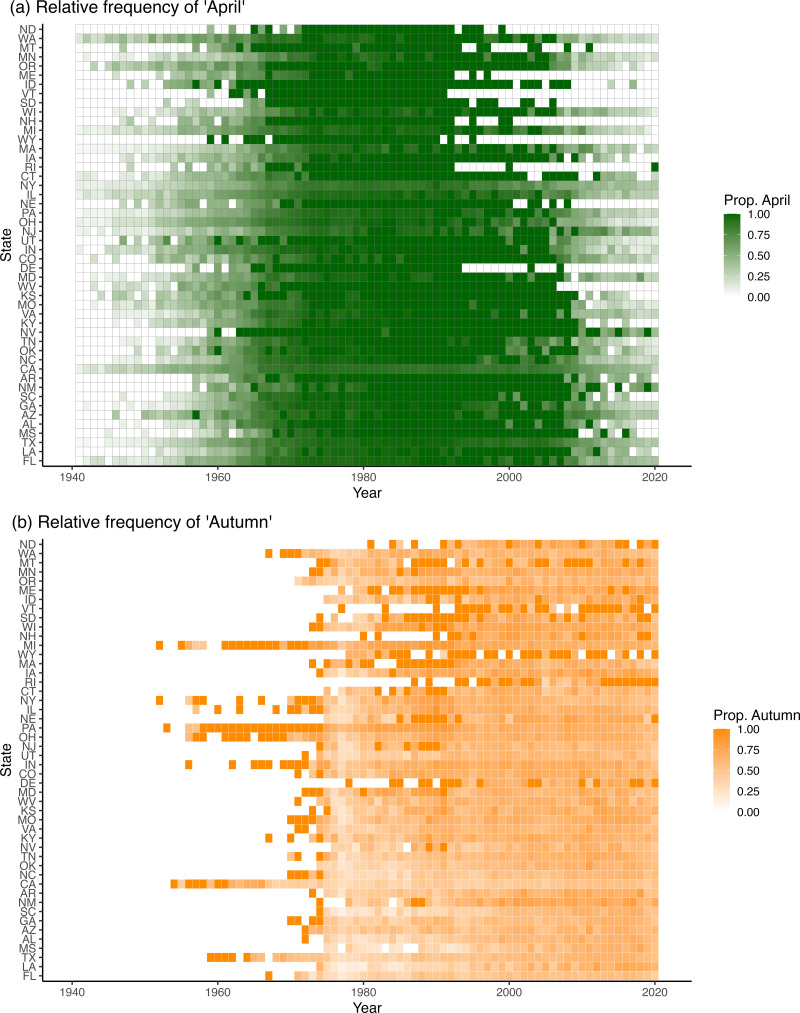
(a). Heat map of proportion of all AMJ girls named April by state and by year. States are arrayed by latitude (top = high latitude). White boxes indicate years when fewer than five girls had an AMJ (or season) name. Note the bubble in April names in essentially all states from ~1970 to ~1990 that began declining in the early 1990s, especially at many mid- to high-latitude states. (b) Heat map of the proportion of girls named Autumn by state and season. Before the mid-1970s, few girls had a season name. A modest bubble in Autumn names occurred during ~1980–1990, but mainly in mid- to high-latitude states.

We determined geographic and climatic correlates of month and of season names, and then inferred patterns of parental choices (Gureckis & Goldstone, 2009; Acerbi & Bentley, 2014). We analysed only traditional English names for months and seasons, because variants (e.g. Apryl for April) are rare or sometimes ambiguous (Supplementary Materials 6). We computed the relative frequencies (by state) of each of the three common month names for girls (April, May, June; Figure 3a). Because such compositional data are non-independent, we analysed the log ratio of *N*_April_/(*N*_April_ + *N*_May_ + *N*_June_), thereby achieving ‘subcompositional coherence’ (Greenacre, 2021). Similarly, we computed the log ratio of Autumn (*N*_Autumn_/(*N*_Autumn_ + *N*_Winter_ + *N*_Spring_ + *N*_Summer_). Because season names were extremely rare before about 1975 (Figure 4b; Supplemental Materials 2, Figure S2d), we restricted seasonal analyses to 1975–2021. We used climate normals (1981–2010) to estimate median dates of the last frost and of first frost by state as indices of the start of spring and of autumn (Figure 2), respectively. However, because frost dates were tightly correlated with latitude, we included only latitude in statistical models to avoid collinearity. Also, because variation in the timing of birth varies with latitude (Martinez-Bakker et al., 2014; Figure S3), thus potentially biasing latitudinal patterns of name popularity, we computed log ratio April births (or log ratio Autumn births) based on the numbers of births per month (season) by state and included these ratios in spatial analyses.

Statistical models accounted for spatial autocorrelation via (Simultaneous Autoregressive Model, ‘SAR,’ Bivand et al., 2021). Spatial weights were generated from the adjacency of states (see Supplementary Materials 1.8).

## Results

### Month names (geographic patterns)

Month names were uncommon for both girls and boys (Rogerson, 2016), but month names were 9.4 times more numerous for girls than boys (0.278 vs. 0.030% of babies of each sex, 1910–2021; Figure 1a). Season names were less common than month names (Figure 1b), but season names were 182 times more numerous for girls than boys (0.135% vs. < 0.001%; Figure 1b). Since 1975, however, season names have increased in popularity (Figure S2).

Parents may choose a month or season name for various reasons unrelated to temporal meaning (e.g. honouring a relative, loved one, celebrity). Interestingly, only 41.6% of a sample of girls with a month name were born in that same month, and only 37.5% of girls with season names were born in that same season. Thus, month (Rogerson, 2016) and season names are only partially associated with birth timing, potentially blurring links between names and seasonal environmental factors. However, as shown below, those links are still strong.

The relative percentage of April names varies substantially among states (range 23.6–77.4%; Figure 3a). Consistent with our first *a priori* prediction, April was the dominant spring-month name in low-latitude CONUS states (Figure 3a,b; note, CONUS is an acronym for Continental United States), where spring starts early (Figure 2a). In contrast, June was dominant in high-latitude states (Figure S4a), where spring starts later (Figure 2b). Indeed, the percentage of April names for the eight states along the southern US border (median = 65.5%, range = 50.1–73.2%) does not overlap with those for the 12 states on the northern border (median = 38.9%, range = 23.4–44.6%).

In a spatial autocorrelation analysis, the log ratio of April declined strongly with log latitude (*p* << 0.0001) but was independent of log mean altitude (*p =* 0.155) and log April births (*p =* 0.53; Supplemental Materials, Table S1). Extra-limital states (Alaska or Hawaii) are striking outliers (Figure 3b, see Supplemental Materials 9).

### Month names (temporal patterns)

Our second *a priori* prediction was that April would have increased in relative frequency in recent years because recent climate warming has advanced the onset of spring (Körner & Basler, 2010). The frequency of April did shift, but non-linearly and wildly over time (Figure 4*a*). Initially, April was uncommon but then became almost the exclusive month name (a name ‘bubble’) from the late 1960s until the end of the twentieth century. Thereafter, April has dropped in relative popularity, especially in northern states (top right Figure 4a).

To examine whether overall latitudinal trends (Figure 3b) persisted in the face of such extreme temporal shifts (Figure 4a), we partitioned the data into three periods (below), based on the frequency of April crossing a 75% frequency threshold. In the initial period (1910–1965), the relative percentage of April was low among states (median = 10.6%). Even so, the log ratio of April declined significantly with log latitude during this period (*p* < 0.0023, Figure S5, Tables S4). During the name bubble in the middle period (~1966 to ~2008), however, virtually all girls with spring-month names were named April (>84.5% in all states, median = 97.4%, Figure S5), and no latitudinal trend was evident (*p* = 0.660, Table S5). During the recent period (2009–2021), April has declined in popularity (median = 31.7%), but the log ratio of April re-established the negative correlation with log latitude (*p* = 0.002, Table S6.). April is currently more popular than in the first period (paired *t*-test, *p* <<< 0.001), consistent with a climate-warming prediction, but whether it continues to drop or stabilises at a relatively elevated level can only be determined in the future.

### Season names

Next, we evaluated our prediction that Autumn would be the most common season name at high latitudes, where autumn weather comes early (Figure 2b) and where deciduous autumn foliage is typically most intensely coloured (Liu et al., 2017). Season names became common only after ~1975 (Figure 4b, Figure S2d) and since then have been relatively stable in popularity (Figure 4b). For 1975–2021, the log ratio of Autumn increased significantly with log latitude (Figure 3e, *p* = 0.009), but not with elevation (*p* = 0.346) or log Autumn births (*p* = 0.386)(Table S2). The mean percentage of Autumn among season names was 70.3% (58.4–86.6%) for the northern border states vs. only 51.8% (45.5–67.4%) for the southern border states – essentially a 36% increase in average popularity.

**Figure 5.**
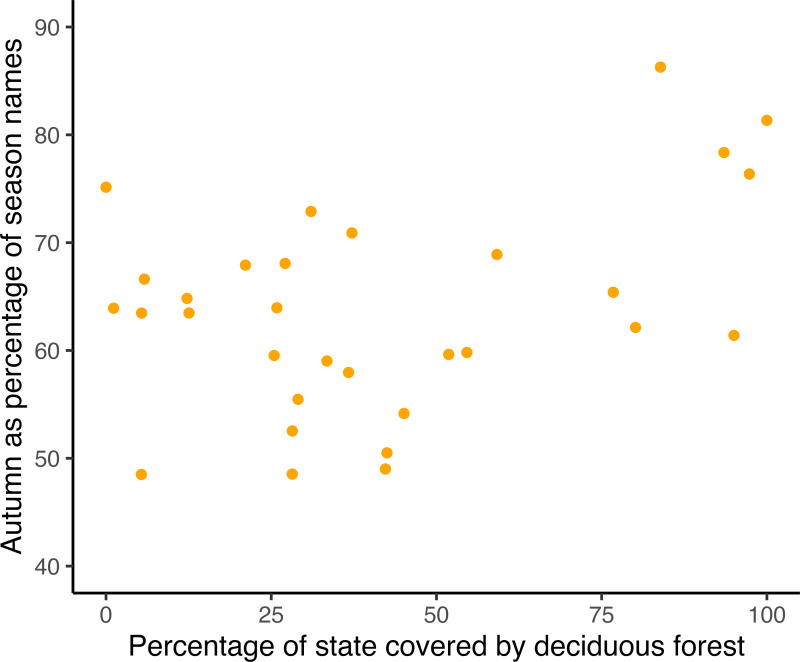
Autumn as a percentage of all season names (by state) increases significantly (p = 0.035, Table S3, seasonalty) with the proportion of that state (30 eastern states only) covered by deciduous shrubs or trees.

The percentage of a state's area that is covered by deciduous foliage is available for 30 eastern states (Liu et al., 2017; Ye & Zhang, 2021). For these states, the log ratio of Autumn was positively correlated with log latitude (*p* << 0.0001) and with the percentage of the state's area currently covered by deciduous or mixed foliage (Figure 5; spatial analysis, *p* = 0.035, Table S3).

The seasonality of climate has diverse ecological and cultural effects (van de Vliert, 2009). For example, unique nicknames in China are relatively uncommon in seasonally demanding climates (He et al., 2021). Consequently, we examined whether the diversity (Shannon index) of month or of season names was correlated with climate seasonality (difference between summer and winter temperatures; Supplemental Materials Figure S6, Table S6). Month-name diversity was unrelated to seasonality (*p =* 0.331), but season-name diversity was conspicuously low in highly seasonal environments (*p =* 0.0009). It is unclear why only the season-name pattern is consistent with an expectation that cultural diversity is constrained by harsh climates (He et al., 2021).

### Hemispheric and continental comparisons

Our month-name predictions were generated specifically for the Northern Hemisphere (NH) but should not hold in English-speaking countries in the Southern Hemisphere (SH), where April, May and June coincide with autumnal, not spring-like weather. Even so, these three names are still the most common month names in Australia (Figure S7). Consequently, we checked whether these month names are nonetheless relatively less popular in the SH than in the NH, as expected based on their oppositional seasonal associations. Any direct comparison of proportional name usage would be confounded by unmeasured cultural, historical and ethnic differences between hemispheres, but the ratio of total month to total season names provides a paired, within-country index of the relative popularity of month vs. season names. Name compilations for major English-speaking counties have limited temporal coverage (Supplementary Materials 2), and we analysed available data for 2000–2020. The month:season ratio varies markedly among countries (Table S7) but is always higher in NH countries (Scotland, Northern Ireland, England and Wales, Canada, CONUS) than in the SH (Australia, New Zealand) (Table S8).

The most popular season name differs between hemispheres or continents. From 2000 to 2020, Autumn was the most popular season name in the USA and Canada, whereas Summer was most popular season name in the UK (except Scotland) and almost exclusively so in Australia and New Zealand (Table S7). The relative popularity of Autumn among continents increases with the continent's proportion of deciduous trees (*r_s_* = 0.850, *p =* 0.015, Table S9). Specifically, North America has a much higher proportion of red-coloured deciduous species than does Europe (Renner & Zohner, 2020), and temperate Australia and New Zealand have relatively few deciduous trees (Dreiss & Volin, 2014). The exceptionally bright reds of North American leaves may reflect relatively high concentrations of anthocyanins and xanthophylls, which provide protection against relatively high autumnal UV radiation in North America compared with Europe (Renner & Zohner, 2020).

## Discussion

Shifts in popularity of baby names are typically viewed through a cultural evolutionary lens. Indeed, given names can be viewed as a cultural ‘product’, and changes in the popularity of such products (e.g. books, music, names) are potentially driven by their intrinsic quality, ‘context-based selection’, drift and negative frequency dependence (Hahn & Bentley, 2003; Berger et al., 2012; Acerbi & Bentley, 2014; Twenge et al., 2016; Newberry & Plotkin, 2022). Given names have limited intrinsic merit (but see Berger et al., 2012; Pitcher et al., 2013; Suire et al., 2019), but their popularity is subject to cultural factors such as context, drift, sexual selection and frequency dependence (Gureckis & Goldstone, 2009; Pitcher et al., 2013; Suire et al., 2019; Newberry & Plotkin, 2022). Here we will argue that – at least for month and season names – context potentially includes not only the social, cultural and political environment of parents, but also their physical and biotic environment.

We proposed and then evaluated three hypotheses relating the popularity of month and season names to geography, climate and foliage. Two were strongly supported. First, April was relatively most popular in low-latitude states (Figure 3a,b) where spring-like weather comes early (Figure 2a). Second, Autumn was most popular in high-latitude states (Figure 3d,e) and in states with a high coverage of deciduous vegetation (Figure 5).

The overall observed covariation of names and environmental factors (Figures 3 and S2.4, S2.5; Tables S1–S3) is consistent with these expectations, and the marked strength of these latitudinal patterns is surprising (Figure 3), especially given that fewer than half of girls with month (season) names were born in that same month (season) (Rogerson, 2016). Alaska and Hawaii are conspicuous outliers for month names (Figure 3b,c). The reason is unclear but might reflect these states’ ‘frontier’ geography (Varnum & Kitayama, 2011), small population size, relatively high overall proportion of immigrants and (for Alaska) the relatively high proportion of immigrants from southern CONUS states (see Supplementary Materials 9). Curiously, these two states are not marked outliers for season names (Figure 3e and f).

Our third prediction was that the popularity of April has increased with climate warming. April is more common now than prior to ~1960 but has been declining in popularity (Figure 4a; Figure S2C). Where and if it stabilises can be determined only in future years.

Some given names have marked boom–bust cycles of popularity (Berger & Le Mens, 2009; Kessler et al., 2012; Xi et al., 2014; Newberry & Plotkin, 2022; Figure S1). Generation-length bubbles appear to be driven by negative frequency-dependent interactions (Newberry & Plotkin, 2022) that involve cultural preferences for novelty vs. commonness (Twenge et al., 2016; Newberry & Plotkin, 2022) or for conformity vs. non-conformity (Acerbi & Bentley, 2014; Denton et al., 2021; Newberry & Plotkin, 2022). In contrast, those names showing geographic signatures potentially suggest the involvement of sustained social, cultural or environmental influences (Fryer & Levitt, 2004; Varnum & Kitayama, 2011; Berger et al., 2012; Xi et al., 2014; Barucca et al., 2015; Pomorski et al., 2016) or sometimes the influence of transient events such as hurricanes or a politician's popularity (Berger et al., 2012; Kułakowski et al., 2016).

Month names illustrate both pulsed and geographic patterns. Most dramatically, April was common at low latitude for decades but then became almost the exclusive spring name around the end of the twentieth century (Figure 4a). Since then, April has declined most strongly at high latitudes (Figure 4a) and has re-established a negative correlation of its popularity with latitude (Figure S5). The end-of-the-century bubble for April coincides with a period of known name instability in the USA (Twenge et al., 2010; Barucca et al., 2015; Pomorski et al., 2016) as well as in the UK (Bush, 2020). It partially overlaps with reproductive years of the Baby Boom Generation (born 1946–1964, Rogerson & Kim, 2005) – a generation known for its disruptive impacts.

Ultimately, direct surveys (Lindsay & Dempsey, 2017) will be needed to establish why parents chose particular baby names, but conducting such surveys on a geographic scale will be logistically challenging and raise privacy concerns. For the present, inferences of individual causation must rely on retrospective analyses of composite data (Acerbi & Bentley, 2014). Causation in such approaches may be obscured (or induced) by drift (Hahn & Bentley, 2003), migration (Rogerson, 2021) or transient fads (Twenge et al., 2016).

Our analyses do not question the dominant role of culture and of social learning in name choice. For one thing, month and season names are too uncommon to serve as models for name choice. Nevertheless, the relative popularity of month and season names of baby girls does correlate strongly with geography and the environment, even though most girls with a month or season name were not born in that month or season. Overall, we interpret these patterns as indirect evidence that environmental factors sometimes override cultural influences in choice of a baby's name.

## Data Availability

All data are publicly available. Sources are listed in the Supplementary Materials.

## Research transparency and reproducibility

To view the code for the main text, please visit https://doi.org/10.1017/ehs.2022.53.

## Supplementary material

For supplementary material accompanying this paper visit https://doi.org/10.1017/ehs.2022.53. click here to view supplementary material
