# Supplementary material for: Signatures of geography, climate and foliage on given names of baby girls
Source: Evol Hum Sci. 2022 Nov 25;4:e56. doi: 10.1017/ehs.2022.53 (PMC10426070; doi:10.1017/ehs.2022.53)
Supplement: Supplementary file 1 [file S2513843X22000536sup.zip › S2513843X22000536sup002.html]

Scripts: Signatures of geography, climate, and foliage on given names of baby girls 


## Table of contents

- README:
- Fig. 1 Lollipop plot of numbers of month & of season names in the USA
- Fig. 2 Choropleth map of last frost & first frost
- Number and percentage of babies with month names (1910 to 2021)
- Number and percentage of babies with season names (1910 to 2021)
- Percentage of girls with month (or season) name that were born in that month (or season)
  - Fig. 3a Choropleth map of April
  - Fig. 3b log April vs. latitude
  - Fig. 3c log ratio April vs. last frost date spring
- Fig. 3d Choropleth map – Autumn
- Fig. 3e log ratio Autumn vs. latitude
- fig. 3f log ratio Autumn vs. first frost date Autumn
- Merge subplots for Fig. 3a:f
- Fig. 4: Heat map of relative proportion of April and of Autumn (by state and year)
- Patterns northern, middle, southern states
- Fig. 5 Autumn popularity versus percentage of states covered by deciduous forest
- Session information

# **Scripts: *Signatures of geography, climate, and foliage on given names of baby girls***

Author

Raymond B. Huey1 & Donald B. Miles2

\*Correspondence: hueyrb@uw.edu & urosaurus@gmail.com

1 Department of Biology, University of Washington, Seattle, Washington, 98195, USA  
2 Department of Biological Sciences, Ohio University, Athens, Ohio, 45701, USA

Packages

```
pacman::p_load(tidyverse, here, vroom, lubridate, usmap, patchwork, vegan, cowplot, ozbabynames, scales, glue, ggtext, usdata, maps, ggalt, ggborderline, sessioninfo,kableExtra)
```

## README:

This file presents scripts used in the main text. A separate Supplementary Material presents additional detail, figures, and tables. The scripts below follow the sequence in the main text. Figure umbers match those in the text. The html file uses code folding: to see the underlying code, click on “Code” in the left margin of each section.

## Fig. 1 Lollipop plot of numbers of month & of season names in the USA

Code

```
# note:  data are imported from Supplemental Materials 2.
tmp <- read_rds(here::here("data_working", "data_national", "NATIONAL-1910-2021-all-names.RDS"))

# add "sex2" -- this reorders M & F so F points are on top of M in graphs
tmp <- tmp |> mutate(
  sex2 = case_when(
    sex == "F" ~ "girl",
    TRUE ~ "boy"
  )
)

N_sex_by_month <- tmp %>%
  filter(month_name == "yes") %>%
  complete(name = month.name) %>%
  group_by(name, sex2) %>%
  summarise(N = sum(number), .groups = "drop") %>%
  tidyr::complete(name, sex2, fill = list(N = 0)) %>%
  # fill in missing years
  filter(sex2 != "NA")

# reorder names in month order
N_sex_by_month$month <- factor(N_sex_by_month$name, levels = c("January", "February", "March", "April", "May", "June", "July", "August", "September", "October", "November", "December"))

# add 'time' as the numerical month (1 = Jan)
# then displace points (vertically) for boy & girl lollipops for plotting
N_sex_by_month <- arrange(N_sex_by_month, sex2, month)
N_sex_by_month$time <- c(seq(0.9, 11.9, by = 1), seq(1.1, 12.1, by = 1))

# draw lollipop plot for month names
lollipop_month <- ggplot(data = N_sex_by_month, aes(y = time, x = N, colour = sex2)) +
  geom_lollipop(horizontal = TRUE, size = 1.5) +
  scale_y_continuous(trans = "reverse", breaks = seq(1, 12, 1), labels = month.name) +
  scale_x_continuous(label = scales::comma, breaks = c(0, 100000, 200000)) +
  theme_classic(base_size = 20) +
  labs(x = "Number of babies", y = "") +
  theme(legend.position = "none") +
  scale_color_manual(values = c("#990099", "#009900")) +
  labs(y = "") +
  plot_annotation(title = "<span style='font-size:18pt'>(a) Month names: <b style='color:#009900;'>girls</b> & <b style='color:#990099;'>boys</b>  </span>", theme = theme(plot.title = element_markdown(lineheight = 2))) +
  theme(legend.position = "none") +
  theme(
    axis.line = element_blank(),
    axis.ticks.y = element_blank()
  )
# lollipop_month

#  Lollipop plot for SEASON names  1910 - 2020

season.name =  c("Spring", "Summer", "Autumn", "Winter")
N_sex_by_season <- tmp %>%
  filter(season_name == "yes") %>%
  complete(name = season.name) %>%
  group_by(name, sex2) %>%
  summarise(N = sum(number), .groups = "drop") %>%
  tidyr::complete(name, sex2, fill = list(N = 0)) %>%
  filter(sex2 != "NA") %>%
  droplevels()

N_sex_by_season$name <- factor(N_sex_by_season$name, levels = c("Spring", "Summer", "Autumn", "Winter"))

# separate time for boy & girl lollipops
N_sex_by_season <- arrange(N_sex_by_season, sex2, name)
N_sex_by_season$time <- c(seq(0.9, 3.9, by = 1), seq(1.1, 4.1, by = 1))

season_name <- c("Spring", "Summer", "Autumn", "Winter")

# draw lollipop plot for SEASON names
lollipop_season <- ggplot(data = N_sex_by_season, aes(y = time, x = N, colour = sex2)) +
  geom_lollipop(horizontal = TRUE, size = 1.3) +
  theme_classic(base_size = 16) +
  scale_y_continuous(trans = "reverse", breaks = seq(1, 4, 1), labels = season_name) +
  scale_x_continuous(label = scales::comma) +
  labs(x = "", y = "", title = "(b) Season names") +
  theme(legend.position = "none") +
  scale_color_manual(values = c("#990099", "#009900")) +
  theme(plot.background = element_rect(fill = "gray95")) +
  theme(legend.position = "none") +
  plot_annotation(title = "<span style='font-size:12pt'>(b) Season names  </span>", theme = theme(plot.title = element_markdown(lineheight = 1.1))) +
  theme(
    axis.line = element_blank(),
    axis.ticks.y = element_blank()
  )

# lollipop_season
# ggsave(here::here("figures/lollipop", "lollipop_b&g_season_names-2022-11-12.pdf"), width = 7.5, height = 5, dpi = 1200)

p_lollipop <- lollipop_month + inset_element(lollipop_season, left = .2, bottom = .05, right = .95, top = .35)

ggsave(here::here("figs_final", "HueyFig1.tiff"), width = 8.6, height = 9.5, dpi = 1200)
ggsave(here::here("figs_final", "HueyFig1c.tiff"), width = 8, height = 7, dpi = 1200)

print(p_lollipop)
```

**Fig. 1.** Lollipop plot of month and of season names by sex (USA).

## Fig. 2 Choropleth map of last frost & first frost

Code

```
# Import spring frost data, then spring, then plot map
# input was txt file, so converted to csv -- otherwise import issues with spacing
# added col names in excel
# <https://protect-eu.mimecast.com/s/8Uv8CqYRRHvNn4WT9RwSQ?domain=ncdc.noaa.gov> saved as NOAA-station-codes-2021-08-13.csv
# file <- "/Users/Huey/Documents/Baby_names_9-21/data_raw/NOAA-station-codes-2021-08-13.csv"
station_codes <- vroom(here::here("data_raw", "NOAA-station-codes-2021-08-13.csv"), show_col_types = FALSE)
# head(station_codes)
# Import frost data SPRING Details in April_frost_2021_12_03.Rmd. using: ann-tmin-prblst-t32Fp50.txt 50% probability date of last 32F occurrence or later, but converted to ann-tmin-prbgsl-t32Fp50.csv because of spacing issues.
date_frost <- vroom(here::here("data_raw", "ann-tmin-prblst-t32Fp50.csv"), show_col_types = FALSE)
colnames(date_frost) <- c("station", "date_flag")

# extract codes by setting up function to split "date_flag" vector
RIGHT <- function(x, n) {
  substring(x, nchar(x) - n + 1)
}
date_frost$flag <- RIGHT(date_frost$date_flag, 1)
# delete last letter of the date vector, this is the flag code above
date_frost$date <- str_sub(date_frost$date_flag, 1, nchar(date_frost$date_flag) - 1)
date_frost$date <- paste0("2010/", date_frost$date) # add 2010 to get date object
date_frost$Date <- ymd(date_frost$date)
# in spring, last frost is May, so filter after June
# date_frost <- date_frost |>filter(date  <  "2010-06-01")
# for meaning of the various flag codes, see
# see https://protect-eu.mimecast.com/s/TW7fCyrllI3oKjnHDdJbH?domain=ncdc.noaa.gov Section VI.Flagslub
date_frost <- date_frost |> filter(flag %in% c("C", "R", "S")) # gets rid of P = provisional and blank = no date (-9999 or equivalent)
date_frost2 <- na.omit(date_frost)

# merge station and frost data
frost_station_date <- date_frost2 |> left_join(station_codes, by = "station")
frost_station_date <- frost_station_date |> drop_na()
frost_station_date$date <- ymd(frost_station_date$date)
frost_station_date$j_date <- yday(frost_station_date$date)
frost_station_date <- frost_station_date |> filter(j_date < 152)
# compute median frost date and median Julian day for spring
median_frost_date_by_state <- frost_station_date |>
  group_by(state) |>
  summarise(
    med_frost_date = median(date, na.rm = TRUE),
    med_julian_frost_date = median(yday(date))
  )

# add Alaska and Hawaii -- for Anchorage, as state data not available. NA frost date for Hawaii (never frosts)
median_frost_date_by_state <- median_frost_date_by_state %>%
  add_row(
    state = "AK",
    med_frost_date = ymd("2010-05-18"),
    med_julian_frost_date = 152
  )

# rename so clear that this is spring frost
median_spring_frost_date_by_state <- median_frost_date_by_state
median_spring_frost_date_by_state <- median_spring_frost_date_by_state |>
  dplyr::rename("med_spring_frost" = med_julian_frost_date)

write_csv(median_spring_frost_date_by_state, here::here("data_working", "median_spring_frost_date_by_state.csv"))

# Import AUTUMN frost data
date_frost <- vroom(here::here("data_raw", "ann-tmin-prbfst-t32Fp50.txt"), show_col_types = FALSE)
colnames(date_frost) <- c("station", "date_flag")
# extract codes by setting up function to split "date_flag" vector
RIGHT <- function(x, n) {
  substring(x, nchar(x) - n + 1)
}
date_frost$flag <- RIGHT(date_frost$date_flag, 1)
date_frost$date <- str_sub(date_frost$date_flag, 1, nchar(date_frost$date_flag) - 1)
# add arbitrary year to create date object
date_frost$date <- paste0("2010/", date_frost$date)
date_frost$Date <- ymd(date_frost$date)
# in spring, last frost is may, so filter after june
date_frost <- date_frost |> filter(date > "2010-06-01")
date_frost <- date_frost |> filter(flag %in% c("C", "R", "S"))
date_frost2 <- na.omit(date_frost)
# merge station data with frost date
frost_station_date <- date_frost2 |> left_join(station_codes, by = "station")
frost_station_date <- frost_station_date |> drop_na()
# compute median frost date and median Julian data by state
median_autumn_frost_date_by_state <- frost_station_date |>
  group_by(state) |>
  summarise(
    med_frost_date = median(Date, na.rm = TRUE),
    med_julian_frost_date = median(yday(Date))
  )
median_autumn_frost_date_by_state <- median_autumn_frost_date_by_state |>
  dplyr::rename("med_autumn_frost" = med_julian_frost_date)
# add data for AK, based on Anchorage
# https://protect-eu.mimecast.com/s/msd9CE9OOHjxOAPc59mAI?domain=garden.org   Sept 23 for 50% at 32°, = 266 julian day
median_autumn_frost_date_by_state <- median_autumn_frost_date_by_state |>
  add_row(
    state = "AK",
    med_frost_date = ymd("2010/09/23"),
    med_autumn_frost = 266
  )

spring_frost <- read_csv(here::here("data_raw", "frost_date_median_state_latitudes_2021-08-18.csv"), show_col_types = FALSE)

spring_frost <- spring_frost |>
  filter(med_julian_frost_date > 10) # gets rid of HI

p_frost_spring <- plot_usmap(data = spring_frost, values = "med_julian_frost_date", color = "white") +
  scale_fill_continuous(
    low = "#deebf7", high = "#084594",
    name = "", limits = c(30, 152),
    breaks = c(30, 60, 90, 120, 150), labels = c("Feb", "Mar", "Apr", "May", "June")
  ) + # blues
  labs(title = "a) Date last frost in spring") +
  theme(plot.title = element_text(size = 15)) +
  theme(
    legend.key.width = unit(.8, "cm"),
    legend.key.height = unit(.3, "cm"),
    legend.text = element_text(size = 10),
    legend.title = element_text(size = 10),
    legend.direction = "horizontal",
    legend.position = "bottom"
  ) +
    theme(legend.position = "bottom")

  theme(legend.position = c(.2, -.12)) # set this low for patchwork, else -.05
```

```
List of 1
 $ legend.position: num [1:2] 0.2 -0.12
 - attr(*, "class")= chr [1:2] "theme" "gg"
 - attr(*, "complete")= logi FALSE
 - attr(*, "validate")= logi TRUE
```

Code

```
# theme(legend.position = c(.2, -.15))   # set this low for patchwork, else -.05
ggsave(here::here("figures", "last_frost_spring.pdf"))
### autumn frost
autumn_frost <- read_csv(here::here("data_working", "Autumn_frost_date_prop.csv"), show_col_types = FALSE)

# frost$region <- tolower(frost$State)
# drop HI as never frosts
autumn_frost <- autumn_frost |> filter(med_julian_frost_date > 10)
# add row for alaska, med_julian_frost_date = 266
ak <- c("AK", "Autumn", 100, .1, "2010-11-09", 266, "Alaska", 32.8066, -86.79113)
x <- rbind(ak, autumn_frost)

# reverse scale by subtrating med_julian_frost_date from 365
# x$rev_date <- 365 - x$med_julian_frost_date
x$med_julian_frost_date <- as.numeric(x$med_julian_frost_date)

# plot Autumn frost dates
p_frost_autumn <- plot_usmap(data = x, values = "med_julian_frost_date", color = "white") +
  scale_fill_continuous(
    low = "#deebf7", high = "#084594",
    name = "", limits = c(244, 365),
    breaks = c(349, 319, 288, 258), labels = c("Dec", "Nov", "Oct", "Sep")
  ) + labs(title = "b) Date first frost in autumn") +
  theme(plot.title = element_text(size = 15)) +
  theme(
    legend.key.width = unit(.8, "cm"),
    legend.key.height = unit(.3, "cm"),
    legend.text = element_text(size = 10),
    legend.title = element_text(size = 10),
    legend.direction = "horizontal"
  ) +
  theme(legend.position = c(.2, -.12))

# combine plots
p_combined <- p_frost_spring + p_frost_autumn
print(p_combined)
```

**Fig. 2.** Choropleth map of **(a)** date of last frost in spring and **(b)** date of first frost in Autumn.

Code

```
ggsave(here::here("figs_final", "HueyFig2a.tiff"), width = 8.6, height = 10, dpi = 1200)
```

## Number and percentage of babies with month names (1910 to 2021)

Code

```
# total N names (girls + boys), in millions, 1910-2021
# label_number_si(accuracy = 0.01)(sum(tmp$number)) # in millions

# number and proportion by name (1910-2021)
sum_month_sex <- tmp %>%
  group_by(sex) %>%
  count(month_name, wt = number, sort = FALSE) %>%
  mutate(Prop = n / sum(n))

n_mon_girls <- format(sum_month_sex[[2, 3]], big.mark = ",")
n_mon_boys <- format(sum_month_sex[[4, 3]], big.mark = ",")
pcnt_mon_girls <- scales::percent(as.numeric(sum_month_sex[2, 4]), accuracy = 0.001)
pcnt_mon_boys <- scales::percent(as.numeric(sum_month_sex[4, 4]), accuracy = 0.001)

# prob girls more likely to have month name
# ratio of girls to boys with month names
# sum_month_sex[[2, 4]] / sum_month_sex[[4, 4]] 

sex <- c("girl", "boy")
total <- c(n_mon_girls, n_mon_boys)
pcnt <- c(pcnt_mon_girls, pcnt_mon_boys)
t_month <- cbind(sex, total, pcnt)

t_month |>
    kbl(caption = "Number and percentage of girls and boys with MONTH names (1910-2020") %>%
    kable_styling(position = "center", font_size = 17)
```

Number and percentage of girls and boys with MONTH names (1910-2020

| sex | total | pcnt |
| --- | --- | --- |
| girl | 480,064 | 0.278% |
| boy | 53,120 | 0.030% |

## Number and percentage of babies with season names (1910 to 2021)

Code

```
sum_season_sex <- tmp %>%
  #     filter(year > 1974) %>%
  group_by(sex) %>%
  count(season_name, wt = number, sort = FALSE) %>%
  mutate(Prop = n / sum(n))

n_season_girls <- format(sum_season_sex[[2, 3]], big.mark = ",")
n_season_boys <- format(sum_season_sex[[4, 3]], big.mark = ",")
pcnt_season_girls <- scales::percent(as.numeric(sum_season_sex[2, 4]), accuracy = 0.001)
pcnt_season_boys <- scales::percent(as.numeric(sum_season_sex[4, 4]), accuracy = 0.001)

total <- c(n_season_girls, n_season_boys)
pcnt <- c(pcnt_season_girls, pcnt_season_boys)
t_season <- cbind(sex, total, pcnt)

# ratio of Ngirls:Nboys with season names
ratio_g2b <- round(sum_season_sex[[2, 4]] / sum_season_sex[[4, 4]], 1)

t_season |>
    kbl(caption = "Number and percentage of girls and boys with SEASON names (1910-2020") %>%
    kable_styling(position = "center", font_size = 15)
```

Number and percentage of girls and boys with SEASON names (1910-2020

| sex | total | pcnt |
| --- | --- | --- |
| girl | 232,689 | 0.135% |
| boy | 1,328 | 0.001% |

## Percentage of girls with month (or season) name that were born in that month (or season)

Code

```
#  This script determines birth month for girls with month or with season names. Then calculates percentage of girls with month names (season) names that were born in that same month (season).
#  For month names, 1910-2020  For season names, 1975-2020.
#  These files are huge and require processing, so the script necessary to do this is commented out below.  The output files are then saved and loaded for use.
####################################################################
# Import DEATH MASTER FILE data
#
# Social Security Death Master File courtesy of SSDMF.INFO.
# from  https://protect-eu.mimecast.com/s/Vbw_CN9DDHp3JnRsKokRd?domain=archive.org
# downloaded 2021-09-11
# there are 3 large (zip) files.  Download each then merge
# Write function to download and process the 3 SSDI files
#  filter for 1910 on for month, 1975 on for season

# month_name <- c("APRIL", "MAY", "JUNE")
# season_name <- c("SPRING", "SUMMER", "AUTUMN", "WINTER")
#
# SSDI_month_names <- function(x) {
#   x <- x %>%
#     dplyr::rename(
#       code = X1,
#       ssn = X2,
#       surname = X3,
#       suffix = X4,
#       givenname = X5,
#       midinit = X6,
#       code2 = X7,
#       DOD = X8,
#       DOB = X9
#     ) %>%
#     dplyr::filter(givenname %in% month_name | givenname %in% season_name) %>%
#     mutate(birth = mdy(DOB), mon = month(birth), year = year(birth)) %>%
#     dplyr::select(givenname, birth, DOB, mon, year)
# }
#
# # READ THE 3 SDMI FILES AND MERGE
# # A FEW ROWS WITH BAD DATES FAIL TO PARSE (23, 19, 16, respectively)
# x <- read_fwf(file = "/Users/Huey/Downloads/ssdm1", fwf_widths(c(1, 9, 20, 4, 15, 15, 1, 8, 8)), show_col_types = FALSE)
# xout <- SSDI_month_names(x)
# y <- read_fwf(file = "/Users/Huey/Downloads/ssdm2", fwf_widths(c(1, 9, 20, 4, 15, 15, 1, 8, 8)), show_col_types = FALSE)
# yout <- SSDI_month_names(y)
# z <- read_fwf(file = "/Users/Huey/Downloads/ssdm3", fwf_widths(c(1, 9, 20, 4, 15, 15, 1, 8, 8)), show_col_types = FALSE)
# zout <- SSDI_month_names(z)
# # merge the 3 files
# xyz <- rbind(xout, yout, zout)
# xyz <- na.omit(xyz)
# xyz$mon <- as.factor(xyz$mon)
# xyz$givenname <- as.factor(xyz$givenname)
# xyzmon <- xyz %>%
#   filter(givenname %in% month_name & year > 1909) %>%
#   droplevels()
# xyzseas <- xyz %>%
#   filter(givenname %in% season_name & year > 1974) %>%
#   droplevels()
# write_csv(xyzmon, here::here("data_working", "xyzmon.csv"))
# write_csv(xyzseas, here::here("data_working", "xyzseas.csv"))

#  PERCENTAGE OF GIRLS WITH A MONTH NAME THAT WERE BORN IN THAT MONTH
xyzmon <- read_csv(here::here("data_working", "xyzmon.csv"), show_col_types = FALSE)
xyzseas <- read_csv(here::here("data_working", "xyzseas.csv"), show_col_types = FALSE)

months_births <- xyzmon |>
  summarise(
    Ntotal = length(year),
    NinMonth = length(year[givenname == "APRIL" & mon == "4"]) +
      length(year[givenname == "MAY" & mon == "5"]) +
      length(year[givenname == "JUNE" & mon == "6"]),
    pcnt_in_month = scales::percent(NinMonth / Ntotal , accuracy = 0.1)) |>
    mutate(Ntotal = scales::comma(Ntotal),
                 NinMonth = scales::comma(NinMonth))

months_births |>
    kbl(caption = "Percentage of girls with AMJ names that were born in that month") |>
    kable_styling(position = "center", font_size = 17)
```

Percentage of girls with AMJ names that were born in that month

| Ntotal | NinMonth | pcnt\_in\_month |
| --- | --- | --- |
| 81,569 | 33,928 | 41.6% |

Code

```
# PERCENTAGE OF GIRLS WITH A SEASON NAME  BORN IN THAT SEASON
#
# group months into seasons
season <- c("WINTER", "SPRING", "SUMMER", "AUTUMN")
bs2 <- xyzseas %>%
  mutate(season = case_when(
    mon == "12" | mon == "1" | mon == "2" ~ "winter",
    mon == "3" | mon == "4" | mon == "5" ~ "spring",
    mon == "6" | mon == "7" | mon == "8" ~ "summer",
    TRUE ~ "autumn"
  ))

seas_births <- bs2 %>%
  summarise(
    Ntotal = length(year),
    NinSeason = length(year[givenname == "AUTUMN" & season == "autumn"]) +
      length(year[givenname == "SPRING" & season == "spring"]) +
      length(year[givenname == "SUMMER" & season == "summer"]) +
      length(year[givenname == "WINTER" & season == "winter"]),
      pcnt_in_season = scales::percent(round(NinSeason/Ntotal, 3)))

# season names
birth_season  <- cbind(seas_births$Ntotal, seas_births$NinSeason, seas_births$pcnt_in_season)

colnames(birth_season) <- c("Total births", "N in season", "Pcnt in season")
birth_season |>
    kbl(caption = "Total girls with SEASON names and percent born in that same SEASON") |>
    kable_styling(position = "center", font_size = 15)
```

Total girls with SEASON names and percent born in that same SEASON

| Total births | N in season | Pcnt in season |
| --- | --- | --- |
| 595 | 223 | 38% |

### Fig. 3a Choropleth map of April

Code

```
tmp <- read_rds(here::here("data_working", "STATE-all-names-2022-06-19.RDS"))
main_month_name <- c("April", "May", "June")

tmp_month <- tmp |>
  filter(sex == "F" & name %in% main_month_name) %>%
  droplevels()

# filter season names for later
season_name <- c("Winter", "Spring", "Summer", "Autumn", "Fall")

tmp_season <- tmp %>%
  filter(sex == "F" & season_name == "yes") %>%
  droplevels()

# compute frequency of April & June, log ratio April
LR_april <- tmp_month %>%
  group_by(state) %>%
  summarise(
    N = sum(number),
    freqApril = sum(number[name == "April"]) / N,
    freqJune = sum(number[name == "June"]) / N,
    logApril = log(sum(number[name == "April"]) / N)
  )

# download (US Census Bureau) population centered latitudes and longitudes for each state
# https://protect-eu.mimecast.com/s/uBI-CQ0JJcLPgDxtRYZo6?domain=census.gov
# requires some fiddling in Excel, save as "state_pop_center.txt"

states <- read_csv(here::here("data_raw", "state_pop_center.csv"), show_col_types = FALSE)
colnames(states) <- c("FP", "state", "population", "latitude", "longitude")
states$state <- state2abbr(states$state)

# add latitude and CONUS variable (yes, not)
LR_april <- LR_april |>left_join(states, by = "state")
LR_april <- LR_april %>%
  mutate(
    CONUS =
      case_when(
        latitude < 25 | latitude > 50 | state == "District of Columbia" ~ "no",
        TRUE ~ "yes"
      )
  )
# delete DC
LR_april <- LR_april |>filter(!state %in% "District of Columbia")

# file from above or just download a saved copy
median_spring_frost_date_by_state <- read_csv(here::here("data_working", "median_spring_frost_date_by_state.csv"), show_col_types = FALSE)


# import last frost date (spring)
LR_april <- left_join(LR_april,
  median_spring_frost_date_by_state %>%
    dplyr::select(state, med_spring_frost),
  by = "state"
)

# Choropleth map OF APRIL
# requires touch up in Illustrator, add line around HI islands show visible
# convert proportion April to percentage

LR_april$pcnt <- 100 * LR_april$freqApril
p_freq_april_all <- plot_usmap(data = LR_april, values = "pcnt", color = "white") +
  scale_fill_gradient(
    low = "#edf8e9", high = "#006d2c", name = "pcnt."
  ) +
  theme(legend.position = "bottom") +
  labs(title = "a) April") +
  theme(plot.title = element_text(size = 14)) +
  theme(
    legend.key.width = unit(.8, "cm"),
    legend.key.height = unit(.3, "cm"),
    legend.text = element_text(size = 10),
    legend.title = element_text(size = 10),
    legend.direction = "horizontal"
  ) +
  theme(legend.position = c(0, -.25)) #
# theme(legend.position = c(.2, -.15)) # set this low for patchwork, else -.05

# ggsave(here::here("figures", "map_Pcnt_april_2021-06-19.pdf"))

print(p_freq_april_all)
```

**Fig.3a.** Choropleth map of proportion of girls named April.

### Fig. 3b log April vs. latitude

Code

```
prop_april_lat <- ggplot(LR_april, aes(latitude, logApril, color = CONUS)) +
  geom_point(size = 2.5) +
  scale_y_continuous(name = "Log ratio April", sec.axis = sec_axis(exp, name = "Prop. April")) +
  scale_color_manual(values = c("black", "#41ab5d")) +
  labs(
    x = "Latitude (°N)",
    title = "b)"
  ) +
  theme_classic(base_size = 14) +
  theme(plot.title = element_text(size = 15)) +
  theme(legend.position = "none") +
  annotate("text", x = 21, y = -1.05, label = "HI") +
  annotate("text", x = 61.3, y = -0.35, label = "AK")

# ggsave("prop_apr_lat_all_years2022-06-19.pdf", height = 6, width = 7)

print(prop_april_lat)
```

**Fig. 3b.** Log ratio of April versus latitude.

### Fig. 3c log ratio April vs. last frost date spring

Code

```
prop_april_frost <- ggplot(LR_april, aes(med_spring_frost, logApril, color = CONUS)) +
  geom_point(size = 2.5) +
  scale_color_manual(values = c("black", "#41ab5d")) +
  scale_y_continuous(name = "Log ratio April", sec.axis = sec_axis(trans = exp, name = "Prop. April")) +
  labs(
    x = "Median date of last frost",
    title = "c)"
  ) +
  theme_classic(base_size = 14) +
  theme(plot.title = element_text(size = 15)) +
  theme(legend.position = "none") +
  scale_x_continuous(
    breaks = c(32, 61, 92, 122, 152),
    labels = c("Feb", "Mar", "Apr", "May", "June")
  ) +
  annotate("text", x = 152, y = -.34, label = "AK")

# ggsave("apr_lat_all_years.pdf", height = 6, width = 6)

print(prop_april_frost)
```

**Fig. 3c.** Log ratio April versus date of last frost in spring. Hawaii does not have frosts so omitted.

## Fig. 3d Choropleth map – Autumn

Code

```
tmp_season <- tmp %>%
  filter(season_name == "yes") # reduce size

LR_autumn <- tmp_season %>%
  group_by(state) %>%
  summarise(
    N = sum(number),
    freqAut = sum(number[name == "Autumn"]) / N,
    freqSum = sum(number[name == "Autumn"]) / N,
    logAut = log(sum(number[name == "Autumn"]) / N)
  )

# delete DC
LR_autumn <- LR_autumn |>filter(!state %in% "DC")
# pop_cent_lat$state <- state2abbr(pop_cent_lat$state)

LR_autumn <- LR_autumn |>left_join(states, by = "state")
LR_autumn <- LR_autumn %>%
  mutate(
    CONUS =
      case_when(
        latitude < 25 | latitude > 50 | state == "DC" ~ "no",
        TRUE ~ "yes"
      )
  )

median_autumn_frost_date_by_state <-  read_csv(here::here("data_working", "median_autumn_frost_date_by_state.csv"), show_col_types = FALSE)
# merge autumn frost datea
LR_autumn <- LR_autumn %>%
  left_join(median_autumn_frost_date_by_state, by = "state")

LR_autumn$pcnt <- 100 * LR_autumn$freqAut # convert freq to pcnt

p_freq_AUTUMN_all <- plot_usmap(data = LR_autumn, values = "pcnt", color = "white") +
  scale_fill_continuous(high = "#983920", low = "#FFE2C1", name = "pcnt.") +
  theme(legend.position = "bottom") +
  # guides(fill = guide_legend(reverse = TRUE)) +
  labs(title = "d) Autumn") +
  theme(plot.title = element_text(size = 14)) +
  theme(
    legend.key.width = unit(.8, "cm"),
    legend.key.height = unit(.3, "cm"),
    legend.text = element_text(size = 10),
    legend.title = element_text(size = 10),
    legend.direction = "horizontal"
  ) +
  theme(legend.position = c(0, -.25)) #

# ggsave(here::here("figures", "map_pcnt_autumn_2022-06-19.pdf"), width = 17.4, height = 14)

print(p_freq_AUTUMN_all)
```

**Fig. 3d.** Choropleth map of proportion of girls named Autumn.

## Fig. 3e log ratio Autumn vs. latitude

Code

```
# layer CONUS so CONUS point is on top
LR_autumn_layer_1 <- LR_autumn[LR_autumn$CONUS == "yes", ]
LR_autumn_layer_2 <- LR_autumn[LR_autumn$CONUS == "no", ]

prop_autumn_lat <- ggplot(LR_autumn, aes(latitude, logAut, color = CONUS)) +
  geom_point(
    data = LR_autumn_layer_1,
    aes(latitude, logAut),
    size = 2.5,
    colour = "#DA784B"
  ) +
  scale_y_continuous(name = "Log ratio Autumn", sec.axis = sec_axis(trans = exp, name = "Prop. Autumn")) +
  geom_point(
    data = LR_autumn_layer_2,
    aes(latitude, logAut),
    size = 2.5,
    colour = "black"
  ) +
  scale_color_manual(values = c("black", "#DA784B")) +
  labs(
    x = "Latitude (°N)",
    title = "e)"
  ) +
  theme_classic(base_size = 14) +
  theme(plot.title = element_text(size = 15)) +
  theme(legend.position = "none") +
  annotate("text", x = 21, y = -1.05, label = "HI") +
  annotate("text", x = 61.5, y = -.25, label = "AK")

# ggsave("prop_autumn_lat_all_years2021-96-19.pdf", height = 6, width = 7)

print(prop_autumn_lat)
```

**Fig. 3e.** log ratio Autumn versus latitude

## fig. 3f log ratio Autumn vs. first frost date Autumn

Code

```
# Autumn by frost
# delete HI as no frost
# layer CONUS so CONUS point is on top
LR_autumn_layer_1 <- LR_autumn[LR_autumn$CONUS == "yes", ]
LR_autumn_layer_2 <- LR_autumn[LR_autumn$CONUS == "no", ]

prop_autumn_frost <- ggplot() +
  geom_point(
    data = LR_autumn_layer_1,
    aes(med_autumn_frost, logAut),
    size = 2.5,
    colour = "#DA784B"
  ) +
  scale_y_continuous(name = "Log ratio Autumn", sec.axis = sec_axis(trans = exp, name = "Prop. Autumn")) +
  geom_point(
    data = LR_autumn_layer_2,
    aes(med_autumn_frost, logAut),
    size = 2.5,
    colour = "black"
  ) +
  labs(
    x = "Median date of first frost",
    title = "f)"
  ) +
  theme_classic(base_size = 14) +
  theme(plot.title = element_text(size = 15)) +
  theme(legend.position = "none") +
  scale_x_continuous(
    breaks = c(274, 305, 335, 366),
    labels = c("Oct", "Nov", "Dec", "Jan")
  ) +
  annotate("text", x = 266, y = -.40, label = "AK")

print(prop_autumn_frost )
```

**Fig. 3f.** Log ratio Autumn versus first frost date in Autumn. Hawaii does not have frosts so omitted.

## Merge subplots for Fig. 3a:f

Code

```
p_all <- (p_freq_april_all + prop_april_lat + prop_april_frost) /
  (p_freq_AUTUMN_all + prop_autumn_lat + prop_autumn_frost)

## ggsave(here::here("figures", "Figure_3_2022-06-18.pdf"))
print(p_all)
```

**Fig. 3.** (a) Choropleth map of log ratio April, (b) log ratio April vs. latitude (population centered), (c) log ratio April vs. median date of last frost in spring, (dD) Choropleth map of log ratio Autumn, (e) log ratio Autumn vs. latitude, (f) log ratio Autumn vs. median date of last frost in spring. N.B.The right y-axis (B,C,E,F) shows proportions that are equivalent to log ratios.

## Fig. 4: Heat map of relative proportion of April and of Autumn (by state and year)

Code

```
### heat maps  April
tmp <- read_rds(here::here("data_working", "STATE-all-names-2022-06-19.RDS"))
nonConus <- c("AK","DC", "HI")
april <- tmp |>
    filter(!state %in% nonConus & month_name == "yes" & sex == "F") |>
    droplevels() %>%
    group_by(state, year) %>%
    summarise(
        total = sum(number),
        nApr  = sum(number[name == "April"]),
        propApr = nApr/total, .groups = "drop")

# add missing years
april <- april |>
    complete(year, nesting(state))
april[is.na(april)] <- 0  # replace NAs with 0

# add latitudes
lats <- read_csv(here::here("data_raw", "pop_center_USA.csv"), show_col_types = FALSE)
lat <- lats |>dplyr::select(STNAME, LATITUDE)
lat <- lat |>mutate(state = as.factor(state2abbr(STNAME)))
april <- left_join(april, lat, by ="state")

# rearrange states by latitude
testlat<- arrange(april, desc(LATITUDE))

#extract state abbreviations
ST3 <- unique(testlat$state)  # list starting with FL to ND
ST2 <- rev(ST3)  #flip order

#convert LATITUDE to factor for plotting, so can use ST3 for labels
aprilFAC <- april |>mutate(latitude = as.factor(LATITUDE))

p_april_years <- ggplot(aprilFAC, aes(year, latitude)) +
    geom_tile(aes(fill = propApr), colour = "grey50") +
    scale_fill_gradient(low = "white", high = "darkgreen") +
    lims(x = c(1940, 2021)) + 
    theme(axis.text.x = element_text(angle = 90)) + 
    labs(title = "(a) Relative frequency of 'April'", x = "Year", y = "State") +
    scale_y_discrete(labels =  ST2) +
    theme_classic(base_size = 14) +
    theme(plot.title = element_text(hjust = -0.08)) +
    labs(fill = "Prop. April")

# ggsave(here::here("figures", "heat_map_Aprilgreen-2021.pdf"))

# Autumn data
# tmp3<- read_rds(here::here("data_working/data_national", "NATIONAL-season-names.RDS"))
tmp <- read_rds(here::here("data_working", "STATE-all-names-2022-06-19.RDS"))

nonConus <- c("AK","DC", "HI")

season_hot <- tmp %>%
    filter(!state %in% nonConus &  sex == "F" & season_name == "yes") |>
    droplevels() |>
    group_by(state, year) %>%
    summarise(
        total = sum(number),
        Naut = sum(number[name == "Autumn"]),
        propAut = Naut/total, .groups = "drop") |>
    complete(year, nesting(state))

season_hot[is.na(season_hot)] <- 0  
# autumn <- tmp3 |>complete(year, nesting(state))
# add lat for each state
lat <- lats |>dplyr::select(STNAME, LATITUDE)
lat <- lat |>mutate(state = as.factor(state2abbr(STNAME)))
testlat <- left_join(season_hot, lat, by ="state")
# rearrange states by latitude
testlat<- arrange(testlat, desc(LATITUDE))

#extract state abbreviations
ST3 <- unique(testlat$state)  # list starting with FL to ND
ST2 <- rev(ST3)  #flip order

# ST2 <- fct_rev(ST)  # state abbreviations ordered
#convert LATITUDE to factor for plotting, so can use ST3 for labels
testlatFAC <- testlat |>mutate(latitude = as.factor(LATITUDE))

p_autumn_years <- ggplot(testlatFAC, aes(year, latitude)) +
    geom_tile(aes(fill = propAut), colour = "white") +
    scale_fill_gradient(low = "white", high = "dark orange") +
    lims(x = c(1940, 2021)) + 
    theme(axis.text.x = element_text(angle = 90)) + 
    labs(title = "(b) Relative frequency of 'Autumn'", x = "Year", y = "State") +
    scale_y_discrete(labels =  ST2) +
    theme_classic(base_size = 14)+
    theme(plot.title = element_text(hjust = -0.08))   +
    labs(fill = "Prop. Autumn")
# p_autumn_years
# ggsave(here::here("figures", "heat_map_Autumn.pdf"))

# combine plots
p_april_autumm_years <- p_april_years /p_autumn_years
p_april_autumm_years
```

**Fig. 4. (A).** Heat map of proportion of AMJ girls named April by state and by year. States are arrayed by latitude (top = high latitude). Gray rectangles indicate years when fewer than five girls had a AMJ or a season name. Note the bubble in April names from ~ 1970 to ~ 2005. Note also that many mid- to high-latitude states had few or no girls with AMJ names after the early 1990s. (**B**) Heat map of proportion of girls named Autumn by state and season. Before the mid-1970s, few girls had a season name. A modest bubble in Autumn names occurred ~ 1980 - 1990, but mainly in mid- to high-latitude states.

Code

```
# ggsave(here::here("figures", "p_april_autumm_years.pdf"),  width = 8, height = 14)
# ggsave(here::here("figures", "p_april_autumm_years_wide.pdf"),  width = 12, height = 14)
```

## Patterns northern, middle, southern states

Code

```
tmp <- read_rds(here::here("data_working", "STATE-all-names-2022-06-19.RDS"))

tmp2 <- tmp %>%
  filter(month_name == "yes") %>%
  droplevels() %>%
  group_by(state) %>%
  summarise(pcnt_April = 100 * sum(number[name == "April"]) / sum(number))

north_state <- c("WA", "ID", "MT", "ND", "MN", "WI", "MI", "OH", "NY", "VT", "NH", "ME")
south_state <- c("CA", "AZ", "NM", "TX", "LA", "MS", "AL", "FL")

tmp3 <- tmp2 %>%
  mutate(
    border =
      case_when(
        state %in% north_state ~ "north",
        state %in% south_state ~ "south",
        TRUE ~ "mid"
      )
  )
tmp3$border <- factor(tmp3$border, levels = c("north", "mid", "south"))


# compute mean % April N & S border states
tmp_out2 <-
  tmp3 |>
  group_by(border) |>
  summarise(
    median_pcnt_april =
      round(median(pcnt_April), 2),
    max_pcnt_April = round(max(pcnt_April), 2),
    min_pcnt_April = round(min(pcnt_April), 2)
  )

tmp_out2|>
  kbl(caption = "Mean, maximum, and minimum proportion Autumn for northern and for southern border states, and for middle states") |>
  kable_styling(position = "center", font_size = 17)
```

Mean, maximum, and minimum proportion Autumn for northern and for southern border states, and for middle states

| border | median\_pcnt\_april | max\_pcnt\_April | min\_pcnt\_April |
| --- | --- | --- | --- |
| north | 38.87 | 44.56 | 23.42 |
| mid | 48.34 | 75.37 | 27.02 |
| south | 65.52 | 73.21 | 50.12 |

Code

```
# season data

tmps <- tmp %>%
  filter(season_name == "yes") %>%
  droplevels() %>%
  group_by(state) %>%
  summarise(prop_Autumn = 100 * sum(number[name == "Autumn"]) /
    sum(number))

tmps <- tmps %>%
  mutate(
    border =
      case_when(
        state %in% north_state ~ "north",
        state %in% south_state ~ "south",
        TRUE ~ "mid"
      )
  )

tmps$border <- factor(tmps$border, levels = c("north", "mid", "south"))

# compute mean % Autumn N & S border states
tmp_outs <-
  tmps %>%
  group_by(border) %>%
  summarise(
    mean_prop_autumn =
      round(median(prop_Autumn), 2),
    max_prop_Autumn = round(max(prop_Autumn), 2),
    min_prop_Autumn = round(min(prop_Autumn), 2)
  )

tmp_outs %>%
  kbl(caption = "Mean, maximum, and minimum proportion Autumn for northern and for southern border states, and for middle states") %>%
  kable_styling(position = "center", font_size = 17)
```

Mean, maximum, and minimum proportion Autumn for northern and for southern border states, and for middle states

| border | mean\_prop\_autumn | max\_prop\_Autumn | min\_prop\_Autumn |
| --- | --- | --- | --- |
| north | 70.30 | 86.57 | 58.37 |
| mid | 61.76 | 80.53 | 28.33 |
| south | 51.84 | 67.43 | 45.48 |

## Fig. 5 Autumn popularity versus percentage of states covered by deciduous forest

Code

```
foliage <- read.csv(here::here("data_working", "Alexis_data_2022-03-21.csv"))

Aug_decid <- ggplot(foliage, aes(100*pcnt_decid, 100*Prop)) +
    geom_point(col = "orange", size = 1.9) +
    ylim(40, 90) +
    xlab("Percentage of state covered by deciduous forest") +
    ylab("Autumn as percentage of season names") +
    theme_classic()

print(Aug_decid)
```

**Fig. 5.** Autumn as a percentage of all season names (by state) increases significantly (P = 0.035, spatial analysis, Supplemental Materials 2, table XX) versus the proportion of that state (data available only for 30 eastern states) covered by deciduous shrubs or trees.

# Session information

─ Session info ────────────────────────  
setting value  
version R version 4.2.2 (2022-10-31)  
os macOS Ventura 13.0.1  
system x86\_64, darwin17.0  
ui RStudio  
language (EN)  
collate en\_US.UTF-8  
ctype en\_US.UTF-8  
tz America/Los\_Angeles  
date 2022-11-16  
rstudio 2022.07.2+576 Spotted Wakerobin (desktop)  
pandoc 2.19.2 @ /Applications/RStudio.app/Contents/MacOS/quarto/bin/tools/ (via rmarkdown)

─ Packages ────────────────────────────  
package \* version date (UTC) lib source  
cowplot \* 1.1.1 2020-12-30 [1] CRAN (R 4.2.0)  
dplyr \* 1.0.10 2022-09-01 [1] CRAN (R 4.2.0)  
forcats \* 0.5.2 2022-08-19 [1] CRAN (R 4.2.0)  
ggalt \* 0.4.0 2017-02-15 [1] CRAN (R 4.2.0)  
ggborderline \* 0.2.0 2022-10-25 [1] CRAN (R 4.2.0)  
ggplot2 \* 3.3.6 2022-05-03 [1] CRAN (R 4.2.0)  
ggtext \* 0.1.2 2022-09-16 [1] CRAN (R 4.2.0)  
glue \* 1.6.2 2022-02-24 [1] CRAN (R 4.2.0)  
here \* 1.0.1 2020-12-13 [1] CRAN (R 4.2.0)  
kableExtra \* 1.3.4 2021-02-20 [1] CRAN (R 4.2.0)  
lattice \* 0.20-45 2021-09-22 [1] CRAN (R 4.2.2) lubridate \* 1.8.0 2021-10-07 [1] CRAN (R 4.2.0)  
maps \* 3.4.1 2022-10-30 [1] CRAN (R 4.2.0)  
ozbabynames \* 0.0.0.9000 2022-11-02 [1] Github (ropenscilabs/ozbabynames@3b5bc67) paletteer \* 1.4.1 2022-08-15 [1] CRAN (R 4.2.0)  
patchwork \* 1.1.2 2022-08-19 [1] CRAN (R 4.2.0)  
permute \* 0.9-7 2022-01-27 [1] CRAN (R 4.2.0)  
purrr \* 0.3.5 2022-10-06 [1] CRAN (R 4.2.0)  
readr \* 2.1.3 2022-10-01 [1] CRAN (R 4.2.0)  
scales \* 1.2.1 2022-08-20 [1] CRAN (R 4.2.0)  
sessioninfo \* 1.2.2 2021-12-06 [1] CRAN (R 4.2.0)  
stringr \* 1.4.1 2022-08-20 [1] CRAN (R 4.2.0)  
tibble \* 3.1.8 2022-07-22 [1] CRAN (R 4.2.0)  
tidyr \* 1.2.1 2022-09-08 [1] CRAN (R 4.2.0)  
tidyverse \* 1.3.2 2022-07-18 [1] CRAN (R 4.2.0)  
usdata \* 0.2.0 2021-06-21 [1] CRAN (R 4.2.0)  
usmap \* 0.6.0 2022-02-27 [1] CRAN (R 4.2.0)  
vegan \* 2.6-4 2022-10-11 [1] CRAN (R 4.2.0)  
vroom \* 1.6.0 2022-09-30 [1] CRAN (R 4.2.0)
