# Supplementary material for: Signatures of geography, climate and foliage on given names of baby girls
Source: Evol Hum Sci. 2022 Nov 25;4:e56. doi: 10.1017/ehs.2022.53 (PMC10426070; doi:10.1017/ehs.2022.53)
Supplement: Supplementary file 1 [file S2513843X22000536sup.zip › S2513843X22000536sup001.html]

Supplementary Materials for ‘Signatures of geography, climate, and foliage on given names of baby girls’


## Table of contents

- README:
- Materials and Methods
  - *1. Examples of evocative uses of month or season names in English literature:*
  - *2. Sources of baby names data:*
  - *3. Sources of data – longitude, latitude, frost dates*:
  - *4. Sources of data – percentage coverage of states by deciduous forests*:
  - *5. Analyses of names:*
  - *6. Are variations on month names rare?*
  - *7. Birth number by month (AMJ) and by season vs. latitude.*
  - *8. Spatial analyses of names*:
  - *9. Alaska and Hawaii as outliers*
  - *10. Inter-continental correlation of month:season ratio and foliage*
- Supplemental figures
  - *1. Popularity of “Liam” over time (USA, UK)*
  - *2. Numbers (total, individual) of month and season names by year*
  - *3. Latitudinal variation in birth timing (USA)*
  - *4. Geographic & climate trends of relative frequency of June & Summer names of girls*
  - *5. Relative frequencies of girls named April during three time periods*
  - *6. Diversity of month and of season names versus climate seasonality*
  - *7. Lollipop plot of numbers of girls & boys with each month & season names in Australia*
  - *1. Spatial analysis of log ratio April*
  - *2. Spatial analysis of log ratio Autumn*
  - *3. Spatial analysis of log ratio Autumn versus a state’s foliage coverage*
  - *4. Spatial analysis of log ratio April vs. latitude (early period)*
  - *5. Spatial analysis of log ratio April vs. latitude (middle period)*
  - *6. Spatial analysis of log ratio April vs. latitude (late period)*
  - *7. Month and season names in several English-speaking countries*
  - \*8. Continent-level correlations between proportion of trees with red foliage versus the ratio (month:season names)
  - 9. Continent-level correlations between proportion of trees with red foliage and relative frequency of Autumn (of season names)
- Session information
- References

# **Supplementary Materials for ‘Signatures of geography, climate, and foliage on given names of baby girls’**

Author

Raymond B. Huey1\* & Donald B. Miles2

1 Department of Biology, University of Washington, Seattle, Washington, 98195, USA  
2 Department of Biological Sciences, Ohio University, Athens, Ohio, 45701, USA   
  
  
  
 \*Correspondence: hueyrb@uw.edu & urosaurus@gmail.com

Packages

```
pacman::p_load(
  tidyverse, here, vroom, lubridate, usmap, patchwork, vegan, cowplot, ozbabynames, scales, glue, ggtext, usdata, maps, ggalt, ggborderline, sessioninfo, pander, tmap, tmaptools, tigris, sf, vroom, formattable, psych, mapproj, paletteer, janitor, tidygeocoder, zoo, spatialreg, spData, ggmap, spdep, maptools, spdplyr, spaMM, stargazer, huxtable, kableExtra, broom
)
```

## README:

This Supplement describes analyses, plots, and tables involving babies with month or with season names. We analyze data from the USA and from some other English-speaking countries (section 2 below).

# Materials and Methods

## *1. Examples of evocative uses of month or season names in English literature:*

“Fall, leaves, fall; die, flowers, away;  
Lengthen night and shorten day;  
Every leaf speaks bliss to me,  
Fluttering from the autumn tree.”  
     – **Emily Brontë**, *Fall Leaves, Fall*

“…it was the spring of hope, it was the winter of our despair…”  
     – **Charles Dickens**, *A Tale of Two Cities*

“A Light exists in Spring  
Not present on the Year  
At any other period -  
When March is scarcely here…”  
     – **Emily Dickinson**, *A Light Exists in Spring*

“No spring nor summer beauty hath such grace  
As I have seen in one autumnal face.”  
     – **John Donne**, *Elegy IX: The Autumnal*

“Delicious autumn! My very soul is wedded to it, and if I were a bird I would fly about the earth seeking the successive autumns.”  
     – **George Eliot**, *Letter to Miss Lewis, Oct. 1, 1841*

“April is the cruelest month, breeding  
Lilacs out of the dead land, mixing  
Memory and desire, stirring  
Dull roots with spring rain.  
Winter kept us warm, covering  
Earth in forgetful snow, feeding  
A little life with dried tubers.”  
     – **T. S. Eliot**, *The Waste Land*

“April…hath put a spirit of youth in every thing.”  
     ― **William Shakespeare**, *Sonnet XCVIII*

“Now is the winter of our discontent…”  
     - **William Shakespeare**, *Richard III*

## *2. Sources of baby names data:*

For the USA we examined Nation-specific (1910-2021) and State-specific (1910-2021) occurrences for baby names for the United States, as downloaded from the Social Security Administration (records current as of 2022-03-07). Each ‘National file’ (years are in separate files) reports sex, given name, and number of occurrences of each name (year of birth was extracted from the file name and added to the data frame). Each ‘State-specific’ file (states separated) reports state, sex, year of birth, given name, and number of occurrences. For privacy reasons, both NATIONAL and STATE files exclude names with fewer than five occurrences per year in any geographic area. Therefore, NATIONAL data are somewhat more complete than are STATE data (50 states + District of Columbia). We analyze data only for 1910-2021 for month names and 1975-2021 for season names (see below).

Under the Freedom of Information Act, we requested (on 2021-11-17 and on 2022-01-04) access to the complete files, but our request (S9H: SSA-2022-001940) was denied on the grounds of privacy. All previously published analyses (by others) also faced this restriction. Session Information is listed at the end of this file.

We used NATIONAL data to discover general trends in given names, but concentrated on STATE data, as our primary interest is establishing geographic/climatic patterns. Limitations of these data sets are outlined in Nuessel (2017).

For Supplemental Fig. 2 (Popularity of **’Liam”** over time) and for analyses involving names from England and Wales, we downloaded data from Office for National Statistics. We accessed Northern Ireland for 2015-2019, and for Scotland for 1998-2019. These data were pooled.

For the Southern Hemisphere, we downloaded data for New Zealand and Australia. New Zealand reports only the top 100 names for 1954-2017, such that uncommon names (e.g., month names) are unlikely to be represented. The R package \*“ozbabynames” is also incomplete for some states/territories. We analyzed data for 2000-2017.

For Canadian provinces, we downloaded data for Alberta for 1990-2020, British Columbia for 1920-2019, Ontario for 1913-2016, and Quebec for 1980-2020. Other provinces reported only a few most popular names and are excluded. Newfoundland and Labrador have data only for top 100 names for 2013-2020 and are excluded

## *3. Sources of data – longitude, latitude, frost dates*:

For the spatial-temporal analyses (see below), we assigned a latitude and longitude to each state, based on the “center of population” from the US Census Bureau for 2010. These central coordinates are weighted by the spatial distribution of people, which is relevant to analyses of the geography of names. Of course, centers of population are not fixed over time (Rogerson and Kim 2005; Rogerson 2021).

For the climate analyses, we indexed onset of spring and of autumn based on frost dates (‘normals’). We downloaded frost data from NOAA for 1981-2010. For an index of onset of spring, we used the date of the “50% probability date of last 32F occurrence or earlier” (‘ann-tmin-prblst-t32Fp50.csv’). For onset of spring, we used “50% probability date of first 32F occurrence or earlier” (‘ann-tmin-prbfst-t32Fp50.csv’)“. These records are for CONUS only. We recorded ‘NA’ for Hawaii (no frosts recorded where people live), and substituted data for Anchorage for AK. For each state, we computed median values.

## *4. Sources of data – percentage coverage of states by deciduous forests*:

A map of the spatial coverage of deciduous and mixed forests for 30 eastern states was copied from figure 3a in ref (24) and digitized courtesy of Alexis Rutschmann.

state <- AL, AR, CT, DE, FL, GA, IL, IN, IA, KY, LA, ME, MD, MA, MI, MN, MS, MO, NH, NJ, NY, NC, OH, PA, RI, SC, TN, VT, VA, WV, WI

latitude <- 33.008097, 35.14258, 41.497001, 39.358946, 27.822726, 33.376825, 41.286759, 40.149246, 41.946066, 37.824499, 30.722814, 44.29995, 39.140769, 42.272291, 42.873187, 45.203555, 32.590954, 38.423798, 43.154858, 40.43181, 41.501299, 35.543075, 40.455191, 40.456756, 41.753609, 34.025176, 35.80809, 44.094874, 37.810313, 38.795594, 43.721933

pcnt\_decid <- 0.4509, 0.367, 0.8012, 0, 0.0535, 0.2905, 0.0115, 0.058, 0.211, 0.2586, 0.4227, 0.935, 0.0539, 0.7675, 0.3721, 0.271, 0.2818, 0.1256, 0.9735, 0.3343, 0.5461, 0.2819, 0.1226, 0.5915, 1, 0.4252, 0.2544, 0.8389, 0.5186, 0.9501, 0.3102

autumn <- 0.3423, 0.3784, 0.4494, 0.3961, 0.3378, 0.3984, 0.5228, 0.5722, 0.4479, 0.4502, 0.3085, 0.4858, 0.4626, 0.4644, 0.7017, 0.468, 0.2755, 0.4648, 0.3613, 0.4136, 0.5338, 0.3789, 0.6907, 0.724, 0.3514, 0.3103, 0.4031, 0.3586, 0.4057, 0.4099, 0.4944

## *5. Analyses of names:*

We analyze month names starting in 1910 and season names starting in 1975 (see below for rationale). For most analyses, we examined only names from ‘CONUS’ states (i.e., continental US states, excluding Washington D.C.), as extralimital states (Alaska, Hawaii) would have undue leverage and are also clear outliers on some analyses. We excluded data for Puerto Rico and US Territories (American Samoa, Guam, Northern Mariana Islands, and U. S. Virgin Islands) because these sites are also geographic outliers and because data are currently available only for 1998-2021.

Our analyses could be based on a month (or season) name as a percentage of all baby names for a given sex. However, especially for rare names, name popularity will be swamped by transient bubbles of popularity in common names (Berger et al. (2012)) as well as by spatial concentrations of diverse ethnic and cultural groups, which sometimes use non-English common names. Therefore, we examine relative percentages a given month name only within the three most common month names (April, May, June) or a given season name as a relative percentage of all season names (Spring, Summer, Autumn, Winter). Because such ‘compositional’ data are non-independent, we focused on April, the dominant month name, and computed and report log ratio as log(NApril/(NApril + NMay + NJune)): this ratio provides ’subcompositional coherence” and is statistically appropriate (Greenacre, 2021). We computed an equivalent log ratio for Autumn, the dominant season name.

## *6. Are variations on month names rare?*

We choose to examine only the frequency of choose to examine only the frequency of traditional month and season names, but variations exist. To determine whether such variations are common, we examined the frequency of variations of April, the most common month name. April constituted 96.3% of all variations on a theme (1950-2020). The script is below.

Code

```
# April as a percent of all  variations on "April".
tmp <- vroom(here::here("data_raw", "names_all_boys_girls_all_years-2021-08-18.csv"), show_col_types = FALSE)
# list of variations on "April."  Uses 'Cs' function from Hmisc, which converts adds quotes to comma separated lists
aprils <- Hmisc::Cs(Abril, Aipril, Aprilete, Aprill, Aprille, Apryl, Apryll, Averel, Averell, Averil, Averill, Averyl, Averyll, Averylle, Avril, Avrill)

tmp <- tmp %>% filter(sex == "F" & year > 1949)
N_april_vars <- tmp %>%
  mutate(april_Names = ifelse(name %in% aprils, "yes", "no")) %>%
  filter(april_Names == "yes") %>%
  summarise(n = sum(number))
N_april <- tmp %>%
  filter(sex == "F" & year > 1949 & name == "April") %>%
  summarise(n = sum(number))
pcnt_April_of_April_variants <- sprintf("%0.2f%%", 100 * N_april / (N_april_vars + N_april))
```

## *7. Birth number by month (AMJ) and by season vs. latitude.*

In the main text, we reported that log ratio April is negatively correlated with latitude, such that relatively more girls named April in the south than in the north. That pattern might reflect parental preference, or it might reflect relatively more April but fewer June births in the north. Importantly, peak birth month does vary with latitude in the USA Martinez-Bakker et al. (2014), and thus a latitude-based birth-month effect could bias the correlation between frequency of April (or Autumn) versus latitude. Consequently, we checked for this potential bias.

We downloaded data on birth month for 2007-2020 Centers for Disease Control and Prevention and then computed log ratio April (based on **N births in April** relative to April-June) and for log ratio Autumn (based on **N births per season**) for each state and included these in our spatial analyses. [Input Query Parameters: “Group By: State; Year; Month; Gender”, “Show Totals: False”, “Show Zero Values: False”, “Show Suppressed: False”, “Calculate Rates Per: , 1,000”, “Population Option: No populations shown because rates were not selected.”]

Log ratio of April births was negatively correlated with latitude (*r* = -0.38, *P* = 0.008, Fig. S3A), thus creating a bias that favors our predicted latitudinal hypothesis. However, the range of percentage of April births among all states is small (31.2% - 32.9%), and log ratio of April births does not contribute significantly to the spatial analyses (see main text).

For season data, log ratio of autumn births was inversely related to latitude (*r* = -0.87, *P* << 0.001, fig. S3B), roughly consistent with Martinez-Bakker et al. (2014), which found that the date of peak birth was inversely related with latitude in three time periods. This induced bias is in the opposite direction of our working hypothesis (Autumn increases with latitude) and with observed data on frequency of Autumn (Fig. 2E). We included log ratio of Autumn births in the spatial analysis (below).

## *8. Spatial analyses of names*:

The patterns in baby names may be influenced by spatial autocorrelation rather than by a direct affect of climate or percentage cover of deciduous trees. Our approach involved spatial analysis of ‘areal’ data. That is, we considered the states to be fixed polygon areas and performed spatial regression analyses that used a proximity matrix as our spatial input. We ran simultaneous autoregression models using the function “spautolm” in the package “*spatialreg*” Bivand and Piras, G. (2021). We created an adjacency matrix using the function “poly2nb” in the package “*spdep*” Bivand (2022). This function creates a list of neighbors from polygons having contiguous boundaries. We used the default “queen = TRUE”, where a single shared boundary point warrants being categorized as contiguous polygons. We used the function “nb2listw” to create a neighbors list with spatial weights. We set the “style” option to “W”, which is a row standardization of the links. The spatially weighted neighbors list was used in the simultaneous autoregression model to control for spatial autocorrelation among the residuals.

## *9. Alaska and Hawaii as outliers*

Alaska and Hawaii are outliers for April (Fig. 2A:C), but much less so for Autumn (Fig. 2D:F). These discrepancies might reflect immigration of parents from other states. For example, perhaps name choice in Alaska might reflect the state in which parents had been raised. Such data are not available. However, as a first approximation of whether between-state migration might influence name choice, we downloaded “mobility” data from the US Census Bureau on 2022-05-19 for the year 2019. This table gives total population (> 1 year old), the new number of residents that came from a different state in the past year, and the number of new residents that came from each other state in the past year.

The discrepancies of Alaska and Hawaii as regards April might in part reflect the relatively small population size of these states (3rd and 11th least populous states) plus their relatively high proportion of immigrants from other states (3rd and 10th, respectively).

Next, to determine whether immigrants were coming from very different latitudes, we looked at the top 5 source states for Alaska and Hawaii. For Alaska, 41.5% of all immigrants from other states came from southern states (Texas, North Carolina, California, Florida, Virginia). For Hawaii, 49.4% of immigrants came from mixed-latitude states (California, Washington, North Carolina, Virginia, Nevada).

In conclusion, why Alaska and Hawaii are outliers for spring names but not season names remains uncertain.

## *10. Inter-continental correlation of month:season ratio and foliage*

The ratios of month to season names for several countries are in Table S6. These were rank correlated against data in (9, 10) for rank order of continents of species with red coloration (North America > Europe > Australia:New Zealand).

# Supplemental figures

## *1. Popularity of “Liam” over time (USA, UK)*

Code

```
# Plot of popularity of "Liam" in USA and in UK over time.
# Source of data in "1.1 Sources of data--baby names"
# data from https://protect-eu.mimecast.com/s/gOjoC2v44TQ3WxjtnBUKSv?domain=ons.gov.uk
liam <- read_csv(here::here("data_raw", "liam.csv"), show_col_types = FALSE)
liam <- pivot_longer(liam, names_to = "country", cols = c("usrank", "ukrank"))
p_liam <- ggplot(liam, aes(year, -value, group = country)) +
  geom_point(aes(shape = country, color = country, fill = country), size = 3) +
  theme_classic(base_size = 14) +
  scale_y_continuous(
    breaks = c(-1, -50, -100),
    labels = c("1", "50", "100")
  ) +
  ggtitle("Popularity of 'Liam' over time") +
  scale_color_manual(values = c("usrank" = "#0072B2", "ukrank" = "#D55E00")) +
  theme(plot.title = element_text(size = 14)) +
  ylab("Rank") +
  xlab("Year") +
  theme(legend.position = "none") +
  theme(plot.title = element_text(hjust = -.12)) +
  annotate("text", label = "UK", x = 2001, y = -5, color = "#D55E00", size = 7) +
  annotate("text", label = "USA", x = 2001.3, y = -100, color = "#0072B2", size = 7) +
  scale_shape_manual(values = c("usrank" = 17, "ukrank" = 16))
# ggsave(here::here("figures", "Liam_2020.pdf"), height = 4.5, width = 6)
print(p_liam)
```

**Fig. 1.** Asychronous and major shifts in the annual ranking of Liam (restricted to top 150 boys) in the USA versus UK demonstrate the fashionable nature of baby names as well as differences in name popularity even between English-speaking countries.

## *2. Numbers (total, individual) of month and season names by year*

Code

```
tmp_month_nat <- read_rds(here::here("data_working/data_national", "NATIONAL-month-N-year-sex.RDS"))

p_month_time <- ggplot(tmp_month_nat, aes(x = year, col = sex, y = N)) +
  theme_classic(base_size = 12) +
  # geom_rect(mapping = aes(xmin = 1950, xmax = 2020, ymin = 0, ymax = 12200), fill = "grey90", col = "white") +
  geom_line(size = 1.1) +
  scale_color_manual(values = c("#009900", "#990099")) +
  scale_y_continuous(limits = c(0, 12500), labels = scales::comma) +
  scale_x_continuous(limits = c(1900, 2022), breaks = seq(1900, 2020, 40)) +
  labs(y = "Number of girls", x = "") +
  ggtitle("A) Month names (total)") +
  theme(
    axis.text = element_text(size = 11),
    axis.title = element_text(size = 12)
  ) +
  theme(plot.title = element_text(size = 12, face = "italic", hjust = 0), plot.title.position = "plot") +
  annotate("text", x = 1925, y = 8120, label = "girls", color = "#009900", size = 4) +
  annotate("text", x = 1925, y = 1185, label = "boys", color = "#990099", size = 4) +
  theme(legend.position = "none")

# season names
tmp_seas_nat <- read_rds(here::here("data_working/data_national", "NATIONAL-season-N-year-sex.RDS"))

p_season_time <- ggplot(tmp_seas_nat, aes(x = year, col = sex, y = N)) +
  theme_classic(base_size = 12) +
  # geom_rect(mapping = aes(xmin = 1975, xmax = 2020, ymin = 0, ymax = 12200),     fill = "grey90", col = "white") +
  geom_line(size = 1.1) +
  scale_color_manual(values = c("#009900", "#990099")) +
  scale_y_continuous(limits = c(0, 12500), labels = scales::comma) +
  scale_x_continuous(limits = c(1900, 2022), breaks = seq(1900, 2020, 40)) +
  labs(y = "", x = "") +
  theme(plot.title = element_text(size = 12, face = "italic", hjust = 0), plot.title.position = "plot") +
  ggtitle("B) Season names (total)") +
  theme(
    axis.text = element_text(size = 11),
    axis.title = element_text(size = 12)
  ) +
  theme(plot.title = element_text(size = 12, face = "italic", hjust = 0), plot.title.position = "plot") +
  theme(legend.position = "none")

# plot of number of individual month names over

tmp_month <- readRDS(here::here("data_working", "girls_month_ALL.RDS"))
AMJ <- c("April", "May", "June") # restrict to April, May, June
tmp_month <- tmp_month %>% filter(name %in% AMJ)
N_AMJ_by_year <- tmp_month %>% count(name, year, wt = number)

p_N_AMJ_years <- ggplot(N_AMJ_by_year, aes(year, n, color = name)) +
  theme_classic(base_size = 12) +
  theme(plot.title = element_text(size = 12, face = "italic", hjust = 0), plot.title.position = "plot") +
  ggtitle("C) Month names (individual)") +
  scale_x_continuous(limits = c(1900, 2022), breaks = seq(1900, 2020, 40)) +
  scale_y_continuous(limits = c(0, 12500), labels = scales::comma) +
  geom_line(aes(color = name), size = 1.1) +
  geom_borderline(linewidth = 1.1, borderwidth = 0.5) +
  scale_color_manual(values = c("#000000", "#009E73", "#D55E00")) +
  scale_shape_manual(values = c(19, 15, 17)) +
  labs(y = "Number of girls", x = "Year") +
  annotate("text", x = 1925, y = 7200, label = "June", color = "#009E73") +
  annotate("text", x = 1993, y = 9000, label = "April", color = "#000000") +
  annotate("text", x = 1925, y = 1440, label = "May", color = "#D55E00") +
  theme(legend.position = "none")

# plot of number of individual season names over
tmp_season <- readRDS(here::here("data_working", "girls_season_ALL.RDS"))
N_season_by_year <- tmp_season %>% count(name, year, wt = number)

p_N_season_year <- ggplot(N_season_by_year, aes(year, n, color = name)) +
  theme_classic(base_size = 12) +
  theme(plot.title = element_text(size = 12, face = "italic", hjust = 0), plot.title.position = "plot") +
  ggtitle("D) Season names (individual)") +
  scale_x_continuous(limits = c(1900, 2022), breaks = seq(1900, 2020, 40)) +
  scale_y_continuous(limits = c(0, 4350), labels = scales::comma) +
  geom_line(aes(color = name), size = 1.1) +
  geom_borderline(linewidth = 1.1, borderwidth = 0.5)  +
  scale_color_manual(values = c("#E69F00", "#009E73", "#000000", "#56B4E9")) +
  # scale_color_manual(values = c("#D55E00", "#009E73", "#56B4E9", "#000000"))
  # scale_shape_manual(values = c(19, 15, 17, 18)) +
  labs(y = "", x = "Year") +
  annotate("text", x = 1984, y = 4000, label = "Autumn", color = "#E69F00") +
  annotate("text", x = 2020, y = 210, label = "Spring", color = "#009E73", size = 3) +
  annotate("text", x = 2010, y = 2665, label = "Summer", color = "#000000") +
  annotate("text", x = 1990, y = 320, label = "Winter", color = "#56B4E9") +
  theme(legend.position = "none")

# combine plots (with patchwork)
p_mon_seas_year <- (p_month_time + p_season_time) / (p_N_AMJ_years + p_N_season_year)
p_mon_seas_year
```

**Fig. S2.** Total number of month **(A)** and of season **(B)** names per year (1910-2020). Number of individual month (C) and season (D) names per year. Note the y-axis scale in (D) differs from the others.

Code

```
# ggsave(here::here("figures", "Names_time_2022-01-19g.pdf"), height = 6, width = 8)
```

## *3. Latitudinal variation in birth timing (USA)*

Code

```
tmp <- read_csv(here::here("data_raw", "Natality, 2007-2020.csv"), show_col_types = FALSE)
# summary(tmp)
tmp2 <- tmp %>%
  filter(Gender == "Female") %>%
  droplevels()

month_names <- c("April", "May", "June")
notConus <- c("Alaska", "District of Columbia", "Hawaii")

tmp3 <- tmp2 %>%
  filter(!State %in% notConus & Month %in% month_names) %>%
  droplevels() # drop nonConus
# Percent of month names (by state) = April
tmp4 <- tmp3 %>%
  group_by(State) %>%
  summarize(
    Nbirths = sum(Births),
    pcnt_April = 100 * sum(Births[Month == "April"]) / Nbirths,
    log_April = log(sum(Births[Month == "April"]) /
      sum(Births)),
    .groups = "drop"
  )
# summary(tmp4$pcnt_April)
# Import pop-centered latitude and merge with tmp4, CONUS
lats <- read_csv(here::here("data_raw", "pop_center_USA.csv"), show_col_types = FALSE)
tmp5 <- left_join(tmp4, lats, by = c("State" = "STNAME"))

# with(tmp5, cor.test(pcnt_April, LATITUDE, method = "pearson"))
# # with(tmp5, cor.test(log_April, LATITUDE, method = "pearson"))
# drop the one low point -- correlation still holds
tmp6 <- tmp5 %>% filter(pcnt_April > 31.5)
# with(tmp6, cor.test(pcnt_April, LATITUDE, method = "pearson"))

# expected 33.0% for april = 30/(30 + 31 + 30) # days in AMJ
p_pcnt_april <- ggplot(tmp5, aes(LATITUDE, pcnt_April)) +
  geom_point(size = 2) +
  geom_hline(yintercept = 32.97, linetype = 2) +
  theme_classic(base_size = 13) +
  labs(
    x = "Centered latitude of state (°N)",
    y = "Percentage of (AMJ) girls born in April",
    title = "(A)"
  ) +
  theme(plot.title = element_text(size = 13)) +
  annotate("text", x = 34.5, y = 33.06, label = "expectation if births at random")
# ggsave(here::here("figures", "p_log_april_lat.pdf"))

# season data
seas1 <- tmp2 %>%
  mutate(season = case_when(
    Month %in% c("December", "January", "February") ~ "winter",
    Month %in% c("March", "April", "May") ~ "spring",
    Month %in% c("June", "July", "August") ~ "summer",
    TRUE ~ "autumn"
  ))

notConus <- c("Alaska", "District of Columbia", "Hawaii")
seas2 <- seas1 %>% filter(!State %in% notConus)

autumn <- seas2 %>%
  group_by(State) %>%
  summarise(
    Nbirths = sum(Births),
    pcnt_autumn = 100 * sum(Births[season == "autumn"]) / Nbirths,
    log_autumn = log(sum(Births[season == "autumn"]) /
      sum(Births)),
    .groups = "drop"
  )
# summary(autumn$pcnt_autumn)
# summary(autumn$log_autumn)
autumn2 <- left_join(autumn, lats, by = c("State" = "STNAME"))
# with(autumn2, cor.test(pcnt_autumn, LATITUDE, method = "spearman"))
# with(autumn2, cor.test(log_autumn, LATITUDE, method = "spearman"))

p_pcnt_autumn <- ggplot(autumn2, aes(LATITUDE, pcnt_autumn)) +
  geom_point(size = 2) +
  geom_hline(yintercept = 24.939, linetype = 2) +
  theme_classic(base_size = 13) +
  labs(
    x = "Centered latitude of state (°N)",
    y = "Percentage of (SSAW) girls born in Autumm",
    title = "(B)"
  ) +
  theme(plot.title = element_text(size = 13)) +
  annotate("text", x = 33, y = 24.9, label = "expectation if random")
pp <- p_pcnt_april + p_pcnt_autumn
pp
```

**Fig. S3.** Latitudinal variation in birth timing. Girls born in April (**A**) or in Autumn (**B**) as a percentage of all girls born versus population centered latitude of each state (CONUS). Dashed line indicate expected percentage of April births if births were random (based on N days/month) with respect to these months. **(B)** Percentage of all-year births occurring in Autumn.

Code

```
# ggsave(here::here("figures", "pcntAprilAutum_lat.pdf"), height = 6, width = 9)
```

## *4. Geographic & climate trends of relative frequency of June & Summer names of girls*

Code

```
tmp <- read_rds(here::here("data_working/data_national", "NATIONAL-all-names.RDS"))
main_month_name <- c("April", "May", "June")
tmp_month <- tmp %>%
  filter(sex == "F" & name %in% main_month_name) %>%
  droplevels()

# filter season names for later
season_name <- c("Winter", "Spring", "Summer", "Autumn", "Fall")
tmp_season <- tmp %>% filter(sex == "F")
tmp_season <- tmp_season %>%
  filter(name %in% season_name) %>%
  droplevels()
# import pop centered lat/long
states <- read_csv(here::here("data_raw", "pop_center_USA.csv"), show_col_types = FALSE)
colnames(states) <- c("id", "state", "population", "latitude", "longitude")

# June PLOTS involving log ratio June, import population-center latitude, import spring-frost date
LR_june <- tmp_month %>%
  group_by(state) %>%
  summarise(
    N = sum(number),
    freqApril = sum(number[name == "April"]) / N,
    freqJune = sum(number[name == "June"]) / N,
    logJune = log(sum(number[name == "June"]) / N)
  )

LR_june$state <- abbr2state(LR_june$state)

# add latitude and CONUS variable (yes, no)
LR_june <- LR_june %>% left_join(states, by = "state")
LR_june <- LR_june %>%
  mutate(
    CONUS =
      case_when(
        latitude < 25 | latitude > 50 | state == "District of Columbia" ~ "no",
        TRUE ~ "yes"
      )
  )
# delete DC
LR_june <- LR_june %>% filter(!state %in% "District of Columbia")

# import last frost date (spring)
median_frost_date_by_state <- read_csv(here::here("data_working", "median_spring_frost_date_by_state.csv"), show_col_types = FALSE)

median_frost_date_by_state$state <- abbr2state(median_frost_date_by_state$state)

median_frost_date_by_state <-   median_frost_date_by_state %>%
  add_row(
    state = "Alaska",
    med_frost_date = ymd("2010-05-18"),
    med_spring_frost = 152
  )

LR_june <- LR_june %>% dplyr::left_join(median_frost_date_by_state, by = "state")

# MAP OF JUNE
LR_june$pcnt <- 100 * LR_june$freqJune
p_freq_june_all <- plot_usmap(data = LR_june, values = "pcnt", color = "white") +
  scale_fill_gradient(
    low = "#edf8e9", high = "#006d2c", name = "pcnt."
  ) +
  theme(legend.position = "bottom") +
  labs(title = "A) June") +
  theme(plot.title = element_text(size = 15)) +
  theme(
    legend.key.width = unit(.7, "cm"),
    legend.key.height = unit(.25, "cm"),
    legend.text = element_text(size = 10),
    legend.title = element_text(size = 10),
    legend.direction = "horizontal"
  ) +
  # theme(legend.position = c(.2, .9)) # for testing legend
  theme(legend.position = c(0, -.25)) # set this low for patchwork, else -.05
# p_freq_june_all
# ggsave(here::here("figures", "map_Pcnt_june_2022-04-05.pdf"))

# Prop June vs. latitude
prop_june_lat <- ggplot(LR_june, aes(latitude, logJune, color = CONUS)) +
  geom_point(size = 2) +
  scale_y_continuous(name = "Log ratio June", sec.axis = sec_axis(exp, name = "Proportion June")) +
  scale_color_manual(values = c("black", "#41ab5d")) +
  labs(
    x = "Latitude (°N)",
    title = "B)"
  ) +
  theme_classic(base_size = 15) +
  theme(plot.title = element_text(size = 15)) +
  theme(legend.position = "none") +
  annotate("text", x = 21, y = -0.59, label = "HI") +
  annotate("text", x = 61, y = -1.5, label = "AK")
# prop_june_lat
# ggsave("prop_apr_lat_all_years2021-12-29.pdf", height = 6, width = 7)

prop_june_frost <- ggplot(LR_june, aes(med_spring_frost, logJune, color = CONUS)) +
  geom_point(size = 2) +
  scale_color_manual(values = c("black", "#41ab5d")) +
  scale_y_continuous(name = "Log ratio June", sec.axis = sec_axis(trans = exp, name = "Proportion June")) +
  labs(
    x = "Median date of last frost",
    title = "C)"
  ) +
  theme_classic(base_size = 15) +
  theme(plot.title = element_text(size = 15)) +
  theme(legend.position = "none") +
  scale_x_continuous(
    breaks = c(32, 61, 92, 122, 152),
    labels = c("Feb", "Mar", "Apr", "May", "June")
  ) +
  annotate("text", x = 152, y = -1.48, label = "AK")

# print(prop_june_frost)

# ggsave("prop_apr_lat_all_years2022-04-05.pdf", height = 6, width = 6)
# Merging plots

p_june <- p_freq_june_all + prop_june_lat + prop_june_frost
# ggsave(here::here("figures", "june_map_lat_frost.pdf"))

# SUMMER
tmp_season <- read_rds(here::here("data_working/data_national", "NATIONAL-season-names.RDS"))

LR_summer <- tmp_season %>%
  group_by(state) %>%
  summarise(
    N = sum(number),
    freqAut = sum(number[name == "Autumn"]) / N,
    freqSum = sum(number[name == "Summer"]) / N,
    logSum = log(sum(number[name == "Summer"]) / N)
  )

# add latitude and CONUS variable (yes, not)
pop_cent_lat <- read_csv(here::here("data_raw", "pop_center_state.csv"), show_col_types = FALSE)
pop_cent_lat$state <- state2abbr(pop_cent_lat$state)

LR_summer <- LR_summer %>% left_join(pop_cent_lat, by = "state")

LR_summer <- LR_summer %>%
  mutate(
    CONUS =
      case_when(
        latitude < 25 | latitude > 50 | state == "DC" ~ "no",
        TRUE ~ "yes"
      )
  )

# read "median_autumn_frost_date_by_state.csv"))
median_autumn_frost_date_by_state <- read_csv(here::here("data_working", "median_autumn_frost_date_by_state.csv"), show_col_types = FALSE)

# delete DC
LR_summer <- LR_summer %>% filter(!state %in% "DC")

LR_summer <- left_join(LR_summer,
  median_autumn_frost_date_by_state %>%
    dplyr::select(state, med_autumn_frost),
  by = "state"
)
# MAP OF SUMMER
LR_summer$pcnt <- 100 * LR_summer$freqSum # convert freq to pcnt
p_freq_SUMMER_all <- plot_usmap(data = LR_summer, values = "pcnt", color = "white") +
  scale_fill_continuous(high = "#983920", low = "#FFE2C1", name = "pcnt") +
  theme(legend.position = "bottom") +
  # guides(fill = guide_legend(reverse = TRUE)) +
  labs(title = "D) Summer") +
  theme(plot.title = element_text(size = 15)) +
  theme(
    legend.key.width = unit(.7, "cm"),
    legend.key.height = unit(.25, "cm"),
    legend.text = element_text(size = 10),
    legend.title = element_text(size = 10),
    legend.direction = "horizontal"
  ) +
  # theme(legend.position = c(.2, .9))
  theme(legend.position = c(0, -.25))
# p_freq_SUMMER_all
# ggsave(here::here("figures", "map_pcnt_summer_2022-04-05.pdf"), width = 17.4, height = 14)

# Plot Prop SUMMER vs. latitude {r fig.height = 6, fig.width = 7}
prop_summer_lat <- ggplot(LR_summer, aes(latitude, logSum, color = CONUS)) +
  geom_point(size = 2) +
  scale_y_continuous(name = "Log ratio Summer", sec.axis = sec_axis(trans = exp, name = "Proportion Summer")) +
  # #41ab5d
  scale_color_manual(values = c("black", "#DA784B")) +
  labs(
    x = "Latitude (°N)",
    title = "E)"
  ) +
  theme_classic(base_size = 15) +
  theme(plot.title = element_text(size = 15)) +
  theme(legend.position = "none") +
  annotate("text", x = 21, y = -0.45, label = "HI") +
  annotate("text", x = 61.5, y = -1.5, label = "AK")

# prop_summer_lat
# ggsave("prop_summer_lat_all_years2022-04-05.pdf", height = 6, width = 7)

# delete HI as no frost,& DC
LR_summer <- LR_summer %>% filter(!state == "HI")
LR_summer <- LR_summer %>% filter(!state == "DC")
# layer CONUS so CONUS point is on top
LR_summer_layer_1 <- LR_summer[LR_summer$CONUS == "yes", ]
LR_summer_layer_2 <- LR_summer[LR_summer$CONUS == "no", ]

prop_summer_frost <- ggplot() +
  geom_point(
    data = LR_summer_layer_1,
    aes(med_autumn_frost, logSum),
    size = 2,
    colour = "#DA784B"
  ) +
  scale_y_continuous(name = "Log ratio Summer", sec.axis = sec_axis(trans = exp, name = "Proportion Summer")) +
  geom_point(
    data = LR_summer_layer_2,
    aes(med_autumn_frost, logSum),
    size = 2,
    colour = "black"
  ) +
  labs(
    x = "Median date of first frost",
    title = "F)"
  ) +
  theme_classic(base_size = 15) +
  theme(plot.title = element_text(size = 15)) +
  theme(legend.position = "none") +
  scale_x_continuous(
    breaks = c(274, 305, 335, 366),
    labels = c("Oct", "Nov", "Dec", "Jan")
  ) +
  annotate("text", x = 266, y = -1.48, label = "AK")

p_all <- (p_freq_june_all + prop_june_lat + prop_june_frost) /
  (p_freq_SUMMER_all + prop_summer_lat + prop_summer_frost)
p_all
```

**Fig. S4.** Geographic and climatic trends of relative frequency of **June** and of **Summer** names of girls. (A) Choropleth map of relative proportion of those girls with spring-month names that are named June. (B) Log ratio of June (equivalent proportion June on right y-axis) is significantly correlated (see text) with population-centered latitude of each CONUS state. Points for Alaska (AK) and Hawaii (HI) are highlighted. (C) Log ratio of June is positively correlated with median date of last frost in spring. [Note: Hawaii does not experience frosts and is absent from panels C and F.] (D) Map of relative proportion of girls with season names that are named Summer (E) Log ratio Summer is inversely correlated with latitude and (F) positively correlated with first frost in autumn.

## *5. Relative frequencies of girls named April during three time periods*

Code

```
month_split <- tmp_month %>%
  mutate(
    period = case_when(
      year < 1966 ~ "1910-1965",
      year > 1965 & year < 2009 ~ "1966-2008",
      TRUE ~ "2009-2020"
    )
  )

April <- month_split %>%
  group_by(state, period) %>%
  summarise(
    N = sum(number),
    freqApr = sum(number[name == "April"]) / N,
    .groups = "drop"
  )

# remove DC, AK, HI
nonConus <- c("AK", "DC", "HI")
April <- April %>%
  filter(!state %in% nonConus) %>%
  droplevels()

# compute ranges of percent April by time period
April_range_by_period <- April %>%
  group_by(period) %>%
  summarise(
    maxApril = max(freqApr),
    medApril = median(freqApr),
    minApril = min(freqApr)
  )
# import latitude

pop_cent_lat <- read_csv(here::here("data_raw", "pop_center_state.csv"), show_col_types = FALSE)

pop_cent_lat$state <- state2abbr(pop_cent_lat$state)

april_lat <- left_join(April, pop_cent_lat, by = "state")
# gives correlation coefficient but not p value
corr_period <- april_lat %>%
  group_by(period) %>%
  summarise(r = cor(latitude, freqApr, method = "pearson"))

early <- cor.test(april_lat$latitude[april_lat$period == "1910-1965"], april_lat$freqApr[april_lat$period == "1910-1965"], method = "pearson")

mid <- cor.test(april_lat$latitude[april_lat$period == "1966-2008"], april_lat$freqApr[april_lat$period == "1966-2008"], method = "pearson")

late <- cor.test(april_lat$latitude[april_lat$period == "2009-2020"], april_lat$freqApr[april_lat$period == "2009-2020"], method = "pearson")

# range of percentages of girls named April for first time period, all high
# summary(april_lat$freqApr[april_lat$period == "1910-1965"])

# range of percentages of girls named April for middle time period, all high
# summary(april_lat$freqApr[april_lat$period == "1966-2008"])

# range of percentages of girls named April for recent time period, all high
# summary(april_lat$freqApr[april_lat$period == "2009-2020"])

#  freq April vs. latitude
p_april_lat <- ggplot(april_lat, aes(latitude, freqApr)) +
  labs(x = "Latitude (°N)", y = "Frequency of April names") +
  geom_point() +
  geom_smooth(method = "lm", se = FALSE, color = "black", formula = y ~ x) +
  theme_classic(base_size = 15) +
  facet_wrap(~period) +
  theme(
    legend.position = "none",
    strip.background = element_rect(colour = "white", fill = "white")
  )
p_april_lat
```

**Fig. S5.** Relative frequency of girls named April versus latitude during three time periods (1950-1974, 1975-1999, 2000-2021). CONUS states only. Spatial patterns are significant for first and third periods (see below).

Code

```
# ggsave(here::here("figures", "p_april_lat_period.pdf"))
```

## *6. Diversity of month and of season names versus climate seasonality*

Code

```
## Shannon index of name diversity vs. seasonality of temperature
# Load data -- The temperatures (monthly averages) are based on data collected
# by weather stations throughout each state during the years 1971 to 2000
# and made available by the NOAA National Climatic Data Center of the
# United States. Downloaded summaries in:
# https://protect-eu.mimecast.com/s/Lx5BCvoqqFZ8qBXTQBjxWa?domain=currentresults.com
seasonality <- read_csv(here::here("data_raw", "USAsummer_winter_temps.csv"), show_col_types = FALSE)
seasonality$abb <- datasets::state.abb
# newrow<- c("WashingtonDC", 1.5, 22.9, 21.4, "DC")  #add DC
# tmp <- rbind(tmp, newrow)
seasonality <- seasonality %>% slice(-c(2, 11)) # cut AK and HI
seasonality$state <- tolower(seasonality$state) # Alabama to alabama
seasonality <- seasonality %>% mutate(
  seasonality = summerC - winterC
)
# write_csv(tmp, here::here("data_working", "USA_seasonality2021-08-16.csv"))

# compute shannon diversity index for month names
# library(vegan) requires reformating d.f.
tmp2 <- read_rds(here::here("data_working", "STATE-all-names-2022-06-19.RDS"))
shan_month <- tmp2 %>%
  group_by(state) %>%
  filter(year > 1949 & sex == "F") %>%
  summarise(
    jan = sum(number[name == "January"]),
    feb = sum(number[name == "February"]),
    mar = sum(number[name == "March"]),
    apr = sum(number[name == "April"]),
    may = sum(number[name == "May"]),
    june = sum(number[name == "June"]),
    july = sum(number[name == "July"]),
    aug = sum(number[name == "August"]),
    sep = sum(number[name == "September"]),
    oct = sum(number[name == "October"]),
    nov = sum(number[name == "November"]),
    dec = sum(number[name == "December"])
  )

nonConus <- c("AK", "DC", "HI")
shan_month <- shan_month %>% filter(!state %in% nonConus)
state <- shan_month$state # save state name
# remove state name for diversity calculation -- needs month counts only
shan_month <- shan_month %>%
  dplyr::select(-state)# remove state name for diversity
# shan_mon_div <- shan_month %>% dplyr::select(2:13)
shan_month <- as.matrix(shan_month)
month_div <- vegan::diversity(shan_month, index = "shannon", MARGIN = 1)

# add back state, diversity, and add long lat
shan_month <- data.frame(shan_month) # convert back to df
shan_month$state <- state
shan_month$shannon <- month_div
# add seasonality 
shan_month <- left_join(shan_month, seasonality, by = c("state" = "abb"))
# add lon lat

pop_cent_lat <- read_csv(here::here("data_raw", "pop_center_USA.csv"), show_col_types = FALSE)
pop_cent_lat <- pop_cent_lat %>% 
    mutate(state = state2abbr(STNAME)) %>% 
    filter(!state  %in% nonConus)
shan_month <- left_join(shan_month, pop_cent_lat, by = c("state"))
# write_csv(shan_month, here::here("data_working", "shan_month.csv"))
p_month_shannon <- ggplot(shan_month, aes(seasonality, shannon)) +
  geom_point() +
  theme_classic(base_size = 16) +
  labs(x = "Seasonality of temperature (°C)", y = "Shannon diversity index (month names)", title = "A)")
###
# season name diversity
shan_season <- tmp_season %>%
  group_by(state) %>%
  filter(year > 1975) %>%
  summarise(
    spring = sum(number[name == "Spring"]),
    summer = sum(number[name == "Summer"]),
    autumn = sum(number[name == "Autumn"]),
    winter = sum(number[name == "winter"])
  )

shan_season <- shan_season %>% filter(!state %in% nonConus)
tmp3shannon <- data.frame(shan_season$spring, shan_season$summer, shan_season$autumn, shan_season$winter)

#shan_season$shannon <- vegan::diversity(shan_season, index = "shannon", MARGIN = 1)
shan_season$shannon <- vegan::diversity(tmp3shannon)

# t$State <- t %>% abbr2state(state)
# t$state <- tolower(t$State)
shan_season <- left_join(shan_season, seasonality, by = c("state" = "abb"))
# write_csv(shan_season, here::here("data_working", "shannonSeason2.csv"))
# with(shannon, cor.test(seasonality, shannon))  #non-spatial correlation
p_season_shannon <- ggplot(shan_season, aes(seasonality, shannon)) +
  geom_point() +
  theme_classic(base_size = 16) +
  labs(x = "Seasonality of temperature (°C)", y = "Shannon diversity index (season names)", title = "B)")

p_shannon <- p_month_shannon + p_season_shannon
p_shannon
```

**Fig. 6. (A)** Diversity of month names (Shannon index) versus seasonality of temperature (monthly max - monthly min). **(B)** Diversity of season names versus seasonality .

## *7. Lollipop plot of numbers of girls & boys with each month & season names in Australia*

Code

```
tmp <- ozbabynames
# Assign factor for whether a month name
month_names <- month.name
tmp <- mutate(tmp, month_name = ifelse(name %in% month_names, "yes", "no"))
tmp$month_name <- factor(tmp$month_name)
tmp2 <- tmp %>%
  filter(month_name == "yes") %>%
  droplevels()

# assign factor for season names  tmp contains month and season names, all years
seas <- c("Winter", "Spring", "Summer", "Autumn") # no "Fall" as no kids named FALL
tmp <- mutate(tmp, season_name = ifelse(name %in% seas, "yes", "no"))
tmp$season_name <- factor(tmp$season_name)
# write_rds(tmp, here::here("data_working/data_national", "NATIONAL-1910-2020-all-names.RDS"))
tmp <- tmp %>% mutate(
  sex2 = case_when(
    sex == "Female" ~ "girl",
    TRUE ~ "boy"
  )
)
N_sex_by_month <- tmp %>%
  dplyr::filter(month_name == "yes") %>%
  complete(name = month.name) %>%
  group_by(name, sex2) %>%
  summarise(N = sum(count), .groups = "drop") %>%
  tidyr::complete(name, sex2, fill = list(N = 0)) %>%
  filter(sex2 != "NA")

# reorder names in month order
N_sex_by_month$month <- factor(N_sex_by_month$name, levels = c("January", "February", "March", "April", "May", "June", "July", "August", "September", "October", "November", "December"))

# use 'displacement' to separate boy & girl lollipops for each month
tmp2 <- arrange(N_sex_by_month, sex2, month)
tmp2$time <- c(seq(0.9, 11.9, by = 1), seq(1.1, 12.1, by = 1))

# draw lolliplot plot for month names
lollipop_month <- ggplot(data = tmp2, aes(y = time, x = N, colour = sex2)) +
  geom_lollipop(horizontal = TRUE, size = 1.5) +
  scale_y_continuous(trans = "reverse", breaks = seq(1, 12, 1), labels = month.name) +
  #     scale_x_continuous(label = scales::comma, breaks = c(0, 100000, 200000)) +
  theme_classic(base_size = 20) +
  labs(x = "Number of babies", y = "") +
  theme(legend.position = "none") +
  scale_color_manual(values = c("#990099", "#009900")) +
  labs(y = "") +
  plot_annotation(title = "<span style='font-size:18pt'>(A) Australian month names: <b style='color:#009900;'>girls</b> & <b style='color:#990099;'>boys</b>  </span>", theme = theme(plot.title = element_markdown(lineheight = 2))) +
  theme(legend.position = "none") +
  theme(
    axis.line = element_blank(),
    axis.ticks.y = element_blank()
  )
# lollipop_month

# ggsave(here::here("figures/lollipop", "OZ-lollipop_b&g_month_names-2022-01-28.pdf"), width = 7, height = 5, dpi = 1200)

# Lollipop plot for SEASON names  1910 - 2020

tmp <- tmp %>% mutate(
  sex2 = case_when(
    sex == "Female" ~ "girl",
    TRUE ~ "boy"
  )
)

N_sex_by_season <- tmp %>%
  filter(season_name == "yes") %>%
  complete(name = seas) %>%
  group_by(name, sex2) %>%
  summarise(N = sum(count), .groups = "drop") %>%
  tidyr::complete(name, sex2, fill = list(N = 0)) %>%
  filter(sex2 != "NA") %>%
  droplevels()

N_sex_by_season$name <- factor(N_sex_by_season$name, levels = c("Spring", "Summer", "Autumn", "Winter"))

# separate boy & girl lollipops for each month
tmp3 <- arrange(N_sex_by_season, sex2, name)
tmp3$time <- c(seq(0.9, 3.9, by = 1), seq(1.1, 4.1, by = 1))

season_name <- c("Spring", "Summer", "Autumn", "Winter")

# draw lolliplot plot for SEASON names
lollipop_season <- ggplot(data = tmp3, aes(y = time, x = N, colour = sex2)) +
  geom_lollipop(horizontal = TRUE, size = 1.3) +
  theme_classic(base_size = 16) +
  scale_y_continuous(trans = "reverse", breaks = seq(1, 4, 1), labels = season_name) +
  scale_x_continuous(label = scales::comma) +
  labs(x = "", y = "", title = "(B) Season names") +
  theme(legend.position = "none") +
  scale_color_manual(values = c("#990099", "#009900")) +
  theme(plot.background = element_rect(fill = "gray95")) +
  # annotate(geom = "text", x = 230000,y = 3.5, label = "girls", color = "#009900") +
  # annotate(geom = "text", x = 31000,y = 7.7, label = "boys", color = "#990099") +
  theme(legend.position = "none") +
  plot_annotation(title = "<span style='font-size:12pt'>(B) Season names  </span>", theme = theme(plot.title = element_markdown(lineheight = 1.1))) +
  theme(
    axis.line = element_blank(),
    axis.ticks.y = element_blank()
  )

# lollipop_season
##  ggsave(here::here("figures/lollipop", "OZ-lollipop_b&g_season_names-2022-01-15.pdf"), width = 7.5, height = 5, dpi = 1200)

lollipop_month + inset_element(lollipop_season, left = .2, bottom = .05, right = .95, top = .35)
```

**Fig. 7.** Lolliplop plot of month and season names in Australia. **(A)** Number of month names for girls and boys **(B)** Number of season names.

Code

```
## ggsave(here::here("figures/lollipop", "OZlollipop_b&g_month_season_names-2022-01-15S.pdf"), width = 8.6, height = 9.5, dpi = 1200)
```

## *1. Spatial analysis of log ratio April*

Code

```
# old numbers
baby.names <- read.csv(here::here("data_working", "New_LR_apr_aut_elev_pop_births_season.csv"))
baby.names <-  baby.names |> rename(
    mean_altitude = mean_m)

# with 2021 data
tmp <- read_rds(here::here("data_working", "STATE-all-names-2022-06-19.RDS"))

main_month_name <- c("April", "May", "June")
tmp_month <- tmp %>%
  filter(sex == "F" & name %in% main_month_name) %>%
  droplevels()

tmp_season <- tmp %>%
  filter(sex == "F" & season_name == "yes") %>%
  droplevels()

# compute frequency of April & June, log ratio April
LR_april <- tmp_month %>%
  group_by(state) %>%
  summarise(
    N = sum(number),
    freqApril = sum(number[name == "April"]) / N,
    freqJune = sum(number[name == "June"]) / N,
    logApril = log(sum(number[name == "April"]) / N)
  )

# download (US Census Bureau) population centered latitudes and longitudes for each state
# https://protect-eu.mimecast.com/s/uBI-CQ0JJcLPgDxtRYZo6?domain=census.gov
# requires some fiddling in Excel, save as "state_pop_center.txt"

states <- read_csv(here::here("data_raw", "state_pop_center.csv"), show_col_types = FALSE)
colnames(states) <- c("FP", "state", "population", "latitude", "longitude")
states$state <- state2abbr(states$state)

# add latitude and CONUS variable (yes, not)
LR_april <- LR_april %>% left_join(states, by = "state")
LR_april <- LR_april %>%
  mutate(
    CONUS =
      case_when(
        latitude < 25 | latitude > 50 | state == "District of Columbia" ~ "no",
        TRUE ~ "yes"
      )
  )
# delete DC
LR_april <- LR_april %>% filter(!state %in% "District of Columbia")

# file from above or just download a saved copy
median_spring_frost_date_by_state <- read_csv(here::here("data_working", "median_spring_frost_date_by_state.csv"), show_col_types = FALSE)


# import last frost date (spring)
LR_april <- left_join(LR_april,
  median_spring_frost_date_by_state %>%
    dplyr::select(state, med_spring_frost),
  by = "state"
)

nonConus <- c("AK", "HI", "DC")
# LR_april <- tmp
# need to replace logApril and log_August so have 2021
april_trans <- LR_april %>%
  filter(!state %in% nonConus) %>%
  droplevels()
baby.names$logApril <- april_trans$logApril

# april_trans done below

foliage <- read.csv(here::here("data_working", "Alexis_data_2022-03-21.csv"))

# corr.test(baby.names$Latitude, baby.names$med_julian_spring_frost_date)
# corr.test(baby.names$mean_m, baby.names$med_julian_spring_frost_date)

##### Import shp file and subset to exclude Alaska, District of Columbia, Hawai'i and Puerto Rico

temp_shapefile <- tempfile()
download.file("https://protect-eu.mimecast.com/s/lL34CG5nni94EygUKGvBQ5?domain=census.gov", temp_shapefile)
unzip(temp_shapefile)
usa <- read_sf("cb_2013_us_state_20m.shp")

CONUS <- usa %>%
  filter(!(NAME %in% c("Alaska", "District of Columbia", "Hawaii", "Puerto Rico")))

Foliage <- usa %>%
  filter(!(NAME %in% c(
    "Alaska", "Arizona", "California", "Colorado",
    "Idaho", "District of Columbia", "Hawaii", "Kansas", "Montana",
    "Nebraska", "Nevada",
    "New Mexico", "North Dakota", "Oklahoma", "Oregon", "Puerto Rico",
    "South Dakota", "Texas", "Utah", "Washington", "Wyoming"
  )))
CONUS_merged <- merge(CONUS, baby.names, by.x = "NAME", by.y = "STATE")
row.names(CONUS_merged) <- CONUS_merged$State
Foliage_merged <- merge(Foliage, foliage, by.x = "STUSPS", by.y = "state")
# View(Foliage_merged)
## Create an adjacency matrix for the states in the US from the shape file.
CONUS_map <- poly2nb(CONUS_merged)
CONUS_nb <- nb2mat(CONUS_map, style = "B")
# class(CONUS_nb)
CONUS_Wnb <- nb2mat(CONUS_map, style = "W")
CONUS.nb2W <- nb2listw(CONUS_map)
April.SAR <- spautolm(logApril ~ log(mean_altitude) + log(Latitude) +
  log_April_births,
data = CONUS_merged, listw = CONUS.nb2W
)
x <- summary(April.SAR)
z <- x$Coef
zprime <- round(z, 4)
zprime %>% 
    kbl(caption = "") %>%
    kable_styling(position = "center", font_size = 17)
```

|  | Estimate | Std. Error | z value | Pr(>|z|) |
| --- | --- | --- | --- | --- |
| (Intercept) | 2.0725 | 5.3383 | 0.3882 | 0.6978 |
| log(mean\_altitude) | 0.0397 | 0.0279 | 1.4228 | 0.1548 |
| log(Latitude) | -1.5471 | 0.3847 | -4.0213 | 0.0001 |
| log\_April\_births | -1.0630 | 1.6879 | -0.6297 | 0.5289 |

Results from a simultaneous autoregressive model with log ratio April as the response variable (CONUS state data, 1910-2021). The model had a value of lambda = 0.56, which is an estimate of the spatial autocorrelation. Lambda was statistically significant (likelihood ratio test value = 8.738, P = 0.0031).

## *2. Spatial analysis of log ratio Autumn*

Code

```
tmp_season <- tmp %>%
  filter(season_name == "yes" & sex == "F" & year > 1974) # reduce size
LR_autumn <- tmp_season %>%
  group_by(state) %>%
  summarise(
    N = sum(number),
    freqAut = sum(number[name == "Autumn"]) / N,
    freqSum = sum(number[name == "Autumn"]) / N,
    logAut = log(sum(number[name == "Autumn"]) / N)
  )

# delete DC
LR_autumn <- LR_autumn %>% filter(!state %in% "DC")
# pop_cent_lat$state <- state2abbr(pop_cent_lat$state)

LR_autumn <- LR_autumn %>% left_join(states, by = "state")
LR_autumn <- LR_autumn %>%
  mutate(
    CONUS =
      case_when(
        latitude < 25 | latitude > 50 | state == "DC" ~ "no",
        TRUE ~ "yes"
      )
  )

median_autumn_frost_date_by_state <-  read_csv(here::here("data_working", "median_autumn_frost_date_by_state.csv"), show_col_types = FALSE)
# merge autumn frost datea
LR_autumn <- LR_autumn %>%
  left_join(median_autumn_frost_date_by_state, by = "state")

autumn_trans <- LR_autumn %>% 
    filter(!state %in% nonConus) %>% 
    droplevels()
baby.names$logAut <- autumn_trans$logAut

Autumn.SAR.noFrost<- spautolm(log_Autumn ~ log(mean_altitude) + log(Latitude) + log_autumn_births,
data = CONUS_merged, listw = CONUS.nb2W)

# summary(Autumn.SAR.noFrost)
x <- summary(Autumn.SAR.noFrost)
z <- x$Coef
zprime <- round(z, 3)
zprime %>% 
    kbl(caption = "") %>%
    kable_styling(position = "center", font_size = 17)
```

|  | Estimate | Std. Error | z value | Pr(>|z|) |
| --- | --- | --- | --- | --- |
| (Intercept) | -5.035 | 1.821 | -2.764 | 0.006 |
| log(mean\_altitude) | -0.015 | 0.016 | -0.943 | 0.346 |
| log(Latitude) | 0.674 | 0.258 | 2.608 | 0.009 |
| log\_autumn\_births | -1.577 | 1.820 | -0.866 | 0.386 |

Results from a simultaneous autoregressive model with log Autumn as the response variable (1975-2021). The model had a value of lambda = 0.31, which is an estimate of the spatial autocorrelation. Lambda was not stastically significant (likelihood ratio test value = 2.027, P = 0.1545).

## *3. Spatial analysis of log ratio Autumn versus a state’s foliage coverage*

Code

```
# Deciduous Cover and Autumn, create the adjacency Matrix
Foliage_map <- poly2nb(Foliage_merged)
Foliage_nb  <- nb2mat(Foliage_map, style="B")
# class(Foliage_nb)
Foliage_Wnb  <- nb2mat(Foliage_map, style="W")

Foliage.nb2W <- nb2listw(Foliage_map)

Autumn.SAR.Foliage<- spautolm(log(Autumn) ~ pcnt_decid + log(Latitude),

                              data = Foliage_merged, listw = Foliage.nb2W)
# summary(Autumn.SAR.Foliage)
x <- summary(Autumn.SAR.Foliage)
z <- x$Coef
```

|  | Estimate | Std. Error | z value | Pr(>|z|) |
| --- | --- | --- | --- | --- |
| (Intercept) | -5.703 | 1.227 | -4.646 | 0.000 |
| pcnt\_decid | -0.252 | 0.119 | -2.110 | 0.035 |
| log(Latitude) | 1.358 | 0.340 | 3.997 | 0.000 |

Results from a simultaneous autoregressive model (for foliage coverage) with log ratio Autumn as the response variable. The model had a value of lambda = 0.31, which is an estimate of the spatial autocorrelation. Lambda was not statistically significant (likelihood ratio test value = 1.503, P = 0.2202).

Code

```
## Analysis of Period Data for April -- setup
April_Prop <- tmp_month %>% 
    filter(!state %in% nonConus)

April_Prop <- April_Prop %>%
  mutate(
    period = case_when(
      year < 1966 ~ "first",
      year > 1965 & year < 2009 ~ "second",
      TRUE ~ "third"
    )
  )
# April_Prop <- left_join(April_Prop, states, by = "state")

# April_Prop <- read.csv(here::here("Dropbox", "Baby_names", "freqApril_lat_frost_by_period_75.csv"))
# April_Prop <- read.csv(here::here("data_working", "freqApril_lat_frost_by_period_75.csv"))

April_Early <- subset(April_Prop, period=="first")
April_Mid <- subset(April_Prop, period=="second")
April_Late <- subset(April_Prop, period=="third")

April_Early <- April_Early %>% 
    group_by(state) %>% 
  summarise(
    N = sum(number),
    freqApril = sum(number[name == "April"]) / N,
    freqJune = sum(number[name == "June"]) / N,
    logApril = log(sum(number[name == "April"]) / N)
  )
April_Early <- left_join(April_Early, states, by = "state")

April_Mid <- April_Mid %>% 
    group_by(state) %>% 
  summarise(
    N = sum(number),
    freqApril = sum(number[name == "April"]) / N,
    freqJune = sum(number[name == "June"]) / N,
    logApril = log(sum(number[name == "April"]) / N)
  )
April_Mid <- left_join(April_Mid, states, by = "state")

April_Late <- April_Late %>% 
    group_by(state) %>% 
  summarise(
    N = sum(number),
    freqApril = sum(number[name == "April"]) / N,
    freqJune = sum(number[name == "June"]) / N,
    logApril = log(sum(number[name == "April"]) / N)
  )
April_Late <- left_join(April_Late, states, by = "state")
#glimpse(April_Early)


# NOTE:  length(April_Late$logApril[April_Late == "-Inf"])
# April_Late has 8 -Inf, so replace logApril for these
# a O score for N april could imply 0 to 4 girls, so use 3
# then compute new log April log(d/N)
# does not replace freqApril
April_Late$logApril <- 
    ifelse(April_Late$freqApril < 0.00001, log(3/April_Late$N), April_Late$logApril)
        

CONUS_April_Early <- merge(CONUS, April_Early, by.x="STUSPS", by.y = 'state')
CONUS_April_Mid <- merge(CONUS, April_Mid, by.x="STUSPS", by.y = 'state')
CONUS_April_Late <- merge(CONUS, April_Late, by.x="STUSPS", by.y = 'state')

# names(CONUS_April_Mid)
# View(CONUS_April_Mid)

CONUS_April_Early_map <- poly2nb(CONUS_April_Early)
CONUS_April_Mid_map   <- poly2nb(CONUS_April_Mid)
CONUS_April_Late_map  <- poly2nb(CONUS_April_Late)

# CONUS_April_Late_map <- poly2nb(CONUS_April_Late) 
CONUS_April_Late_Wnb  <- nb2mat(CONUS_April_Late_map, style="B")
# class(CONUS_nb)
CONUS_April_Early_Wnb  <- nb2mat(CONUS_April_Early_map, style="W")
CONUS_April_Mid_Wnb  <- nb2mat(CONUS_April_Mid_map, style="W")
CONUS_April_Early_nb2W <- nb2listw(CONUS_April_Early_map)
CONUS_April_Mid_nb2W <- nb2listw(CONUS_April_Mid_map)
CONUS_April_Late_nb2W <- nb2listw(CONUS_April_Late_map)
```

## *4. Spatial analysis of log ratio April vs. latitude (early period)*

Code

```
April.SAR.April_Early <- spautolm(logApril ~ log(latitude),
                            data = CONUS_April_Early, listw = CONUS_April_Early_nb2W)
#summary(April.SAR.April_Early)
x <- summary(April.SAR.April_Early)
z <- x$Coef
```

|  | Estimate | Std. Error | z value | Pr(>|z|) |
| --- | --- | --- | --- | --- |
| (Intercept) | 7.5469 | 3.2225 | 2.3420 | 0.0192 |
| log(latitude) | -2.6829 | 0.8789 | -3.0525 | 0.0023 |

Results from a simultaneous autoregressive model for the *early period* (1910-1965) with log ratio April as the response variable. The model had a value of lambda = 0.71, which is an estimate of the spatial autocorrelation. Lambda was statistically significant (likelihood ratio test value = 20.703, P = 0).

## *5. Spatial analysis of log ratio April vs. latitude (middle period)*

Code

```
April.SAR.April_Mid <- spautolm(logApril ~ log(latitude),
                                  data = CONUS_April_Mid, listw = CONUS_April_Mid_nb2W)
x <- summary(April.SAR.April_Mid)
z <- x$Coef
```

|  | Estimate | Std. Error | z value | Pr(>|z|) |
| --- | --- | --- | --- | --- |
| (Intercept) | 0.0393 | 0.1734 | 0.2265 | 0.8208 |
| log(latitude) | -0.0208 | 0.0473 | -0.4391 | 0.6606 |

Results from a simultaneous autoregressive model with log ratio April for the *middle period* (1966-2006) as the response variable. The model had a value of lambda = 0.27, which is an estimate of the spatial autocorrelation. Lambda was statistically significant (likelihood ratio test value = 2.342, P = 0.126).

## *6. Spatial analysis of log ratio April vs. latitude (late period)*

Code

```
# this period includes
April.SAR.April_Late <- spautolm(logApril ~ log(latitude),
                                data = CONUS_April_Late, listw = CONUS_April_Late_nb2W)

x <- summary(April.SAR.April_Late)
z <- x$Coef
```

|  | Estimate | Std. Error | z value | Pr(>|z|) |
| --- | --- | --- | --- | --- |
| (Intercept) | 9.1769 | 3.4121 | 2.6895 | 0.0072 |
| log(latitude) | -2.8723 | 0.9310 | -3.0852 | 0.0020 |

Results from a simultaneous autoregressive model with log ratio April for the *late period* (2007-2011) as the response variable. The model had a value of lambda = 0.41, which is an estimate of the spatial autocorrelation. Lambda was statistically significant (likelihood ratio test value = 4.733, P = 0.0296).

## *7. Month and season names in several English-speaking countries*

Month and Season names in English-speaking countries. This script computes the N of girls with month or with season names, and computes the ratio of month to season names (see maintext). Each country is a separate script, then merged. Canada reports by province, so these were merged before grouping with other countries.

Code

```
# set up function to tabulate summary stats
# x is the df for the country
CountrySummary <- function(x, y) {
  x %>%
    filter(month_name == "yes" | season_name == "yes") %>%
    summarise(
      country = y,
      N_names = sum(number),
      N_month_names = sum(number[month_name == "yes"]),
      N_season_names = sum(number[season_name == "yes"]),
      ratio = N_month_names / N_season_names,
      sumAMJ = sum(number[name == "April" | name == "May" | name == "June"]),
      pcntAMJ = 100 * sumAMJ / sum(N_month_names),
      pcntAutumn = 100 * (sum(number[name == "Autumn"])) / N_season_names,
      sumAutumn = sum(number[name == "Autumn"]),
      sumSummer = sum(number[name == "Summer"]),
      pcntSummer = 100 * sum(number[name == "Summer"]) / N_season_names
    )
}

# USA
folder <- "/Users/Huey/Documents/Baby_names_9-21/data_raw/namesnational/"
filelist <- list.files(path = folder, pattern = "*.txt", full.names = TRUE)
US <- vroom::vroom(filelist, col_names = FALSE, delim = , show_col_types = FALSE, id = "path") # loads and merges all TXT files
US$year <- as.numeric(unlist(str_sub(US$path, 66, 69)))
US <- US %>% dplyr::select(-path)
colnames(US) <- c("name", "sex", "number", "year") # add column names
US <- US %>% mutate_if(is.character, as.factor)
#   start in 2000
US <- US %>%
  filter(year > 1999 & sex == "F") %>%
  droplevels()

month_names <- month.name
US <- mutate(US, month_name = ifelse(name %in% month.name, "yes", "no"))
season_names <- c("Winter", "Spring", "Summer", "Autumn") 
US <- mutate(US, season_name = ifelse(name %in% season_names, "yes", "no"))
USAout <- CountrySummary(US, "United States")

# ozbabynames
oz <- ozbabynames %>% filter(year > 1999 & sex == "Female")
oz <- oz %>% rename(number = count)
oz <- oz %>%
  mutate(
    month_name = ifelse(name %in% month_names, "yes", "no"),
    season_name = ifelse(name %in% season_names, "yes", "no")
  ) %>%
  filter(month_name == "yes" | season_name == "yes") %>%
  droplevels()

OZout <- CountrySummary(oz, "Australia")

# Scotland -- very complete, has as few as 1 girl per name!
SC <- read_csv(here::here("data_raw", "Scotland.csv"), show_col_types = FALSE)
SC <- mutate(SC,
  month_name = ifelse(name %in% month_names, "yes", "no"),
  season_name = ifelse(name %in% season_names, "yes", "no")
)
SCout <- CountrySummary(SC, "Scotland")

# UK-Wales
## years in columns so need to sum across years
tmp <- read_csv(here::here("data_raw", "UKzero.csv"), show_col_types = FALSE) 
tmpyr <- tmp %>% dplyr::select(Name:"2000") # 2000 to 2020 tmpyr$name <- as_factor(tmpyr$Name) 
tmpyr <- tmpyr %>% replace(is.na(.), 0) # some NA value
UK <- tmpyr %>% mutate(number = rowSums(across(where(is.numeric)))) # total names during period
library("stringi")
UK$name <- stri_trans_totitle(UK$Name) # all caps to title format 
UK <- mutate(UK, month_name = ifelse(name %in% month_names, "yes", "no"), season_name = ifelse(name %in% season_names, "yes", "no"))
UKout <- CountrySummary(UK, "UK-Wales")

# Northern-Ireland}
# NI <- read_csv(here::here("data_raw", "NorthernIreland06-20.csv"), show_col_types = FALSE)
NI <- read_csv(here::here("data_raw", "NI_9720.csv"), show_col_types = FALSE) 
NI <- NI %>% rename("name" = "Name/Year") 
colnames(NI) <- c("name", "1997":"2020") 
NI <- NI %>% dplyr::select(c("name", "2000":"2020")) 
NI <- NI %>% mutate(number = rowSums(across(where(is.numeric)))) 
NI$name <- as_factor(NI$name)
NI <- mutate(NI, month_name = ifelse(name %in% month_names, "yes", "no"), season_name = ifelse(name %in% season_names, "yes", "no"))
NIout <- CountrySummary(NI, "Northern Ireland")

# Canada-merged}
BC <- read_csv(here::here("data_raw", "bc-popular-girls-names.csv"), show_col_types = FALSE) 
BC <- BC %>% dplyr::select(c(Name, `2000`:`2019`)) %>% mutate(number = rowSums(across(where(is.numeric)))) # total names during period 
BC$name <- stri_trans_totitle(BC$Name) # all caps to title format 
BC <- mutate(BC, month_name = ifelse(name %in% month_names, "yes", "no"), season_name = ifelse(name %in% season_names, "yes", "no"))
BCout <- CountrySummary(BC, "BC")

Alb <- read_csv(here::here("data_raw", "Alberta-baby-names-frequency.csv"), show_col_types = FALSE)
Albs <- Alb %>% dplyr::filter(sex == "Girl" & year > 1999) %>% mutate( month_name = ifelse(name %in% month_names, "yes", "no"), season_name = ifelse(name %in% season_names, "yes", "no")) %>% dplyr::filter(month_name == "yes" | season_name == "yes") %>% droplevels()
Albout <- CountrySummary(Albs, "Alb")

Ont <- read_csv(here::here("data_raw", "Ontario2022.csv"), show_col_types = FALSE) 
colnames(Ont) <- c("year", "name", "number") 
Ont$name <- stri_trans_totitle(Ont$name)  
Ont <- mutate(Ont, month_name = ifelse(name %in% month_names, "yes", "no"), season_name = ifelse(name %in% season_names, "yes", "no"))
Ontout <- CountrySummary(Ont, "Ont")

Que <- read_csv(here::here("data_raw", "Quebec2022.csv"), show_col_types = FALSE)
Que$name <- stri_trans_totitle(Que$name) 
Que <- Que %>% mutate( month_name = ifelse(name %in% month_names, "yes", "no"), season_name = ifelse(name %in% season_names, "yes", "no") ) %>% dplyr::select(c(name, `2000`:`2020`, month_name, season_name)) %>% mutate(number = rowSums(across(where(is.numeric)))) %>% filter(month_name == "yes" | season_name == "yes") %>% droplevels()
Queout <- CountrySummary(Que, "Que")

## New Brunswick -- top 10 only,might be more but couldn't find.
## Manitoba -- top 10 only skip
## Yukon --top 21 max skip
## PEI fewer than 15 -- skip
## Saskatchewan -- top 20 only, skip
## Newfoundland gives only rankings of top 100, skip
# Nova Scotia top 100 only -- 2003-2017 website says earlier but can't download

## https://protect-eu.mimecast.com/s/kDAhCgLYYsJyKgkSNx4P_l?domain=opendata.gov.nl.ca

## 2022-02-12 the file below no longer accessible

NS <- read_csv(here::here("data_raw", "NovaScotia2022.csv"), show_col_types = FALSE)
NS$name <- stri_trans_totitle(NS$name) 
NS <- mutate(NS, month_name = ifelse(name %in% month_names, "yes", "no"), season_name = ifelse(name %in% season_names, "yes", "no"))
NS <- NS %>% filter(month_name == "yes" | season_name == "yes" & sex == "F")
NSout <- CountrySummary(NS, "NS")

# Canada-merged
Canada <- rbind(BCout, NSout, Albout, Ontout, Queout) 
Canout <- Canada %>% summarise(country = "Canada", N_names = sum(N_names), N_month_names = sum(N_month_names), N_season_names = sum(N_season_names), ratio = sum(N_month_names) / sum(N_season_names), sumAMJ = sum(sumAMJ), pcntAMJ = 100 * sum(sumAMJ) / sum(N_month_names), pcntAutumn = 100 * sum(sumAutumn) / sum(N_season_names), sumAutumn = sum(sumAutumn), sumSummer = sum(sumSummer), pcntSummer = 100 * sum(sumSummer) / sum(N_season_names) )

# New-Zealand}
NZ <- read_csv(here::here("data_raw", "NZ-top100-2000-2020.csv"), show_col_types = FALSE)
NZ <- mutate(NZ, month_name = ifelse(name %in% month_names, "yes", "no"), season_name = ifelse(name %in% season_names, "yes", "no")) 

NZ <- NZ %>% filter(month_name == "yes" | season_name == "yes")

NZout <- CountrySummary(NZ, "New Zealand")

# merge-all-countries}
all_cntr <- rbind(Canout, USAout, SCout, NIout, UKout,  OZout, NZout) # 
# all_countries$ratio <- all_countries$ratio 
# knitr::kable(all_cntr) 
coluse <- c("country", "N_month_names", "N_season_names", "ratio", "pcntAMJ", "pcntAutumn", "pcntSummer") 
all_cntr <- all_cntr %>% select(all_of(coluse)) 
all_cntr2 <- all_cntr %>% 
    mutate(country = country, 
                 N_month_names = scales::comma(N_month_names), 
                 N_season_names = scales::comma(N_season_names), 
                 ratio = round(ratio, 1), 
                 pcntAMJ = round(pcntAMJ, 1), 
                 pcntAutumn = round(pcntAutumn, 1), 
                 pcntSummer = round(pcntSummer, 1) )

colnames(all_cntr2 ) <- c("Country", "N month names", "N season names", "ratio", "% AMJ", "% Autumn", "% Summer")

all_cntr2[7,5] <- 0

knitr::kable(all_cntr2, booktabs = TRUE,caption = "Continental patterns", align = c("crcccc")) %>% 
# all_cntr2 %>% 
    # kbl(booktabs = TRUE) %>% 
    pack_rows("North America", 1, 2) %>%
    pack_rows("Europe", 3, 5) %>%
    pack_rows("Australia, New Zealand", 6,7) %>%
    kableExtra::kable_styling(font_size = 13)
```

Continental patterns

| Country | N month names | N season names | ratio | % AMJ | % Autumn | % Summer |
| --- | --- | --- | --- | --- | --- | --- |
| **North America** | | | | | | |
| Canada | 17,328 | 7,849 | 2.2 | 99.2 | 50.6 | 45.3 |
| United States | 45,101 | 135,455 | 0.3 | 83.7 | 61.0 | 31.6 |
| **Europe** | | | | | | |
| Scotland | 1,325 | 2,028 | 0.7 | 97.0 | 15.2 | 81.5 |
| Northern Ireland | 7,715 | 7,813 | 1.0 | 100.0 | 53.0 | 41.5 |
| UK-Wales | 5,682 | 26,359 | 0.2 | 99.1 | 17.1 | 79.7 |
| **Australia, New Zealand** | | | | | | |
| Australia | 379 | 4,919 | 0.1 | 98.4 | 0.8 | 98.0 |
| New Zealand | 0 | 1,472 | 0.0 | 0.0 | 0.0 | 100.0 |

Comparison of month and of season names in English-speaking countries (2000-2020, see Materials and Methods). Ratio is the number of month:season names. % AJM is the percentage of girls with any month name that were named April, May, or June: most girls with month names have one of these names. % Autumn and % Summer refer to the percentage of all season names that are these season names. Summer is more common in Europe and the Southern Hemisphere, whereas Autumn is more common in North America. The most common season name for each country is in boldface. Note: New Zealand reports only the top 100 names, so the 0 score for N month names is likely an underestimate.

## \*8. Continent-level correlations between proportion of trees with red foliage versus the ratio (month:season names)

Code

```
# rank correlation for red color ("a") vs. ratio ("b")
# red colors are ranks of percentages of red species for Canada & USA, Northern Ireland & Scotland UK &Australia + New Zealand
# ratio and %Autumn (see below) are from Table S7, herein
# rankings for based on DOI: 10.1111/nph.15900 for North America and Europe and  DOI: 10.1081/E-ENRL-120047447 for Australia and New Zealand
test1 <- tibble( a = c(1.5, 1.5, 4, 4, 4, 6.6, 6.6), b = c(2.2, 0.3, 0.7, 1, 0.2, 0.1, 0) )
cor_ratio <- with(test1, cor.test(a, b, method = "spearman"))
tidy(cor_ratio)
```

| estimate | statistic | p.value | method | alternative |
| --- | --- | --- | --- | --- |
| -0.756 | 98.3 | 0.0493 | Spearman's rank correlation rho | two.sided |

## 9. Continent-level correlations between proportion of trees with red foliage and relative frequency of Autumn (of season names)

Code

```
test2 <- tibble( a = c(1.5, 1.5, 4, 4, 4, 6.6, 6.6), b = c(50.6, 61.4, 15.2, 53, 17.1, .8, 0))

cor_autumn <- with(test2, cor.test(a,b, method = "spearman"))
tidy(cor_autumn)
```

| estimate | statistic | p.value | method | alternative |
| --- | --- | --- | --- | --- |
| -0.85 | 104 | 0.0153 | Spearman's rank correlation rho | two.sided |

# Session information

─ Session info ────────────────────────  
setting value  
version R version 4.2.2 (2022-10-31)  
os macOS Ventura 13.0.1  
system x86\_64, darwin17.0  
ui RStudio  
language (EN)  
collate en\_US.UTF-8  
ctype en\_US.UTF-8  
tz America/Los\_Angeles  
date 2022-11-16  
rstudio 2022.07.2+576 Spotted Wakerobin (desktop)  
pandoc 2.19.2 @ /Applications/RStudio.app/Contents/MacOS/quarto/bin/tools/ (via rmarkdown)

─ Packages ────────────────────────── package \* version date (UTC) lib source cowplot \* 1.1.1 2020-12-30 [1] CRAN (R 4.2.0)  
dplyr \* 1.0.10 2022-09-01 [1] CRAN (R 4.2.0)  
forcats \* 0.5.2 2022-08-19 [1] CRAN (R 4.2.0)  
ggalt \* 0.4.0 2017-02-15 [1] CRAN (R 4.2.0)  
ggborderline \* 0.2.0 2022-10-25 [1] CRAN (R 4.2.0)  
ggplot2 \* 3.3.6 2022-05-03 [1] CRAN (R 4.2.0)  
ggtext \* 0.1.2 2022-09-16 [1] CRAN (R 4.2.0)  
glue \* 1.6.2 2022-02-24 [1] CRAN (R 4.2.0)  
here \* 1.0.1 2020-12-13 [1] CRAN (R 4.2.0)  
kableExtra \* 1.3.4 2021-02-20 [1] CRAN (R 4.2.0)  
lattice \* 0.20-45 2021-09-22 [1] CRAN (R 4.2.2)  
lubridate \* 1.8.0 2021-10-07 [1] CRAN (R 4.2.0)  
maps \* 3.4.1 2022-10-30 [1] CRAN (R 4.2.0)  
ozbabynames \* 0.0.0.9000 2022-11-02 [1] Github (ropenscilabs/ozbabynames@3b5bc67)  
paletteer \* 1.4.1 2022-08-15 [1] CRAN (R 4.2.0)  
patchwork \* 1.1.2 2022-08-19 [1] CRAN (R 4.2.0)  
permute \* 0.9-7 2022-01-27 [1] CRAN (R 4.2.0)  
purrr \* 0.3.5 2022-10-06 [1] CRAN (R 4.2.0)  
readr \* 2.1.3 2022-10-01 [1] CRAN (R 4.2.0)  
scales \* 1.2.1 2022-08-20 [1] CRAN (R 4.2.0)  
sessioninfo \* 1.2.2 2021-12-06 [1] CRAN (R 4.2.0)  
stringr \* 1.4.1 2022-08-20 [1] CRAN (R 4.2.0)  
tibble \* 3.1.8 2022-07-22 [1] CRAN (R 4.2.0)  
tidyr \* 1.2.1 2022-09-08 [1] CRAN (R 4.2.0)  
tidyverse \* 1.3.2 2022-07-18 [1] CRAN (R 4.2.0)  
usdata \* 0.2.0 2021-06-21 [1] CRAN (R 4.2.0)  
usmap \* 0.6.0 2022-02-27 [1] CRAN (R 4.2.0)  
vegan \* 2.6-4 2022-10-11 [1] CRAN (R 4.2.0)  
vroom \* 1.6.0 2022-09-30 [1] CRAN (R 4.2.0)

## References

Berger, Jonah, Eric T. Bradlow, Alex Braunstein, and Yao Zhang. 2012. “From Karen to Katie: Using Baby Names to Understand Cultural Evolution.” *Psychological Science* 23 (10): 1067–73. https://protect-eu.mimecast.com/s/ueCCC32RRFvKo8BTqDugbJ?domain=doi.org.

Bivand, R. 2022. “R Packages for Analyzing Spatial Data: A Comparative Case Study with Areal Data.” *Geographical Analysis* 0: 1–31. https://protect-eu.mimecast.com/s/1WRFC4RmmTx5EjosxVRjR2?domain=doi.org.

Bivand, R., and Piras, G. 2021. *Spatialreg: Spatial Regression Analysis. R Package Version 1.2- 1*. https://protect-eu.mimecast.com/s/qPgCC59nnH193nviO8ekTJ?domain=r-spatial.github.io/.

Martinez-Bakker, Micaela, Kevin M. Bakker, Aaron A. King, and Pejman Rohani. 2014. “Human Birth Seasonality: Latitudinal Gradient and Interplay with Childhood Disease Dynamics.” *Proceedings of the Royal Society B: Biological Sciences* 281 (1783): 20132438. https://protect-eu.mimecast.com/s/GWWLC6RooTJm73DS6Bqrwk?domain=doi.org.

Nuessel, Frank. 2017. “A Note on Popular Baby Names on the Social Security Website: An Important Onomastic Resource.” *Names* 65 (1): 45–50. https://protect-eu.mimecast.com/s/uSJxC7L88sYN2PqCW2K5Np?domain=doi.org.

Rogerson, Peter A. 2021. “Historical Change in the Large-Scale Population Distribution of the United States.” *Applied Geography* 136 (November): 102563. https://protect-eu.mimecast.com/s/qujOC8688hgqWJrt1EQruE?domain=doi.org.

Rogerson, Peter A., and Daejong Kim. 2005. “Population Distribution and Redistribution of the Baby-Boom Cohort in the United States: Recent Trends and Implications.” *Proceedings of the National Academy of Sciences* 102 (43): 15319–24. https://protect-eu.mimecast.com/s/ShDgC9Q66fjqAZ1cEQs3ZU?domain=doi.org.
